# Mucociliary Respiratory Epithelium Integrity in Molecular Defense and Susceptibility to Pulmonary Viral Infections

**DOI:** 10.3390/biology10020095

**Published:** 2021-01-29

**Authors:** Adivitiya, Manish Singh Kaushik, Soura Chakraborty, Shobi Veleri, Suneel Kateriya

**Affiliations:** 1 Laboratory of Optobiology, School of Biotechnology, Jawaharlal Nehru University, New Delhi 110067, India; adivitiya1989@gmail.com (A.); manishskaushik@jnu.ac.in (M.S.K.); sourachakraborty.02@gmail.com (S.C.); 2 Drug Safety Division, ICMR-National Institute of Nutrition, Hyderabad 500007, India; shobi.veleri@icmr.gov.in

**Keywords:** mucociliary clearance, lung cilia, goblet cells, mucus, respiratory diseases, microbial infections, coronavirus

## Abstract

**Simple Summary:**

Mucociliary clearance constitutes an innate lung defense mechanism that is primarily driven by ciliated cells. Respiratory mucus traps pathogens entering the airways, and lung cilia propel them outward via their coordinated directional motion. Thus, damage to the component(s) of this apparatus will hamper its smooth functioning. Here, we update the cellular and molecular machinery that constitutes and regulates mucociliary clearance (MCC). We also describe several respiratory diseases arising due to genetic or acquired molecular shortcomings in the MCC. The past few decades have seen the emergence of novel viruses that inflame and damage the respiratory tract. Coronaviruses have been observed to disrupt the ciliated epithelium and abolish its integrity. Bearing in mind the havoc created by the ongoing pandemic, we outline the significance of the ciliated respiratory epithelium in defense against such microbial infections. We have predicted protein interaction networks depicting severe acute respiratory syndrome coronavirus-2 (SARS-CoV-2)-manifested implications on the molecular machinery regulating mucociliary clearance. Several proteins involved in the network were found to interact with SARS-CoV-2 viral proteins upon host infection. This review also emphasizes the importance of the proper management and surveillance of respiratory health in the elderly and patients with chronic respiratory diseases so that they do not bear the impact of a severe or lethal infection.

**Abstract:**

Mucociliary defense, mediated by the ciliated and goblet cells, is fundamental to respiratory fitness. The concerted action of ciliary movement on the respiratory epithelial surface and the pathogen entrapment function of mucus help to maintain healthy airways. Consequently, genetic or acquired defects in lung defense elicit respiratory diseases and secondary microbial infections that inflict damage on pulmonary function and may even be fatal. Individuals living with chronic and acute respiratory diseases are more susceptible to develop severe coronavirus disease-19 (COVID-19) illness and hence should be proficiently managed. In light of the prevailing pandemic, we review the current understanding of the respiratory system and its molecular components with a major focus on the pathophysiology arising due to collapsed respiratory epithelium integrity such as abnormal ciliary movement, cilia loss and dysfunction, ciliated cell destruction, and changes in mucus rheology. The review includes protein interaction networks of coronavirus infection-manifested implications on the molecular machinery that regulates mucociliary clearance. We also provide an insight into the alteration of the transcriptional networks of genes in the nasopharynx associated with the mucociliary clearance apparatus in humans upon infection by severe acute respiratory syndrome coronavirus-2.

## 1. Introduction

Life depends on the availability of oxygen in humans. The human respiratory system is an intricate interface for breathing and gaseous exchange. Coupled with the circulatory system, it ensures a constant oxygen supply to the living tissues and removes carbon di oxide to properly sustain metabolic homeostasis. The air is filtered, warmed, and humidified during inhalation before traversing the trachea and the dichotomous respiratory airways. This air stream comes close, within one cell layer barrier, to the blood stream and thereby exposes the body, via the respiratory tract, to a myriad of foreign agents like pathogenic bacteria, viruses, gaseous, and particulate matter present in the inhaled air. However, the respiratory tract has sophisticated defenses to guard against these potentially noxious agents. Mucociliary clearance (MCC) is the innate lung defense machinery used to capture and clear inhaled foreign agents [1,2]. The airway mucosa is lined by a pseudostratified epithelium where the ciliated and secretory cells provide the primary barrier during attack by a foreign agent [3,4]. The mucosa is covered by the airway surface layer (ASL). It includes a protective layer of mucus to ensnare the inhaled foreign elements and microbes, as well as a periciliary layer (PCL) for the lubrication of respiratory airways to facilitate efficient ciliary movement that drives effective mucus expulsion. In conjunction, the metachronal beating of cilia on the epithelial surface generates a wave-like motion that propels the pathogens and particulates trapped within the mucus layer of the airways outward and towards the nose or mouth for their elimination via coughing or swallowing. Thus, in healthy individuals, an effective MCC system coordinates the mucus formation (that traps dust and pathogens to be propelled out) and the clearance of foreign agents mediated by cilia. Consequently, a weakened or collapsed defense due to the malfunctioning of one or more components of the MCC apparatus permits the development of chronic respiratory diseases that may be genetic or acquired, such as chronic obstructive pulmonary disease (COPD), asthma, cystic fibrosis (CF), and primary ciliary dyskinesia (PCD). Furthermore, smoking, air pollution, and the inhalation of dust or chemical particles (occupational hazards) also increase the risk for developing respiratory infections and airway diseases, thereby contributing to the global burden of pulmonary diseases [5,6]. Additionally, socio-ecological changes in the current century have resulted in the emergence of virulent pathogens, such as the coronaviruses, that target the respiratory tract. Among them, the infection by severe acute respiratory syndrome coronavirus-2 (SARS-CoV-2) has evolved into the pandemic coronavirus disease-19 (COVID-19). The current situation poses an enormous challenge for managing the pulmonary diseases with an unprecedented dimension. In this context, we review the important components of the human respiratory system with a specific focus on the MCC apparatus, highlighting the critical role of cilia and mucus to effectively clear pathogenic agents entering the lungs. We also discuss the health hazards associated with a dysfunctional mucociliary escalator. Finally, we present an insight into the impact of SARS-CoV-2 infection on molecules (proteins) associated with or involved in the mucociliary clearance system of the host.

## 2. Organization and Components of the Human Respiratory System

**Figure 1:**
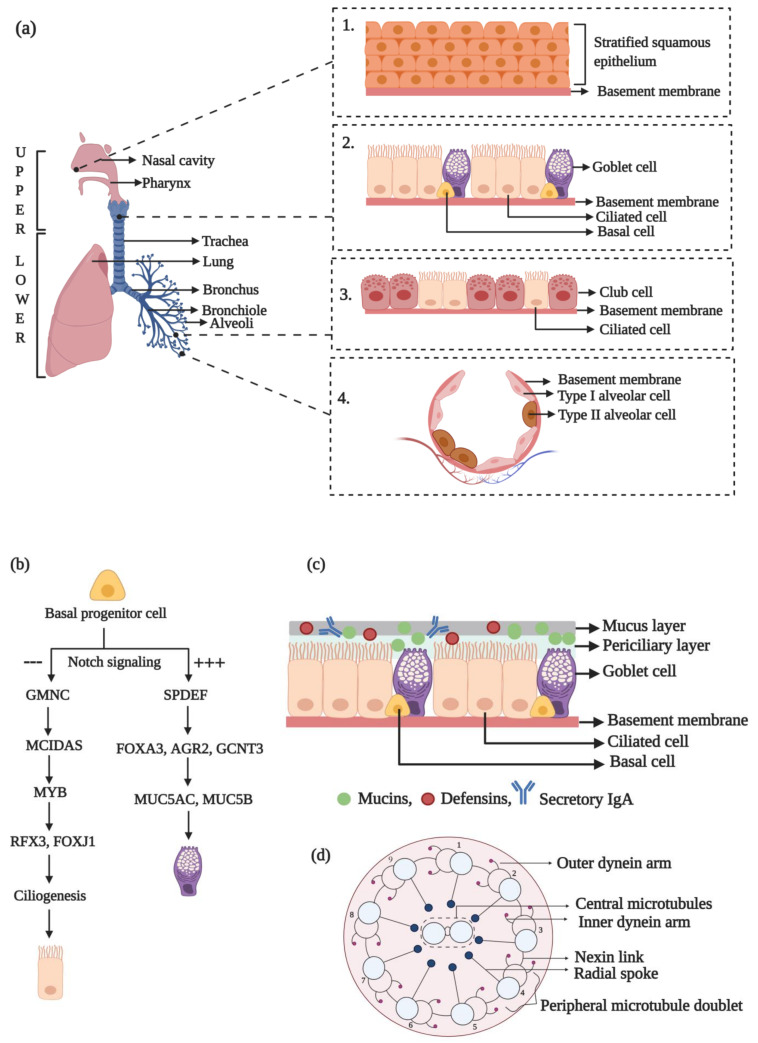
A pictorial visualization of the morphology and development of the components of the respiratory epithelium. (**a**) The human respiratory system comprises of the upper and lower respiratory tracts that collectively function to carry out gaseous exchange and also protect against air-borne infections. The nasal vestibule in the upper respiratory tract is lined by stratified squamous epithelium (Inset 1). It transitions into a pseudostratified columnar epithelial mucosa with ciliated and goblet cells lying on a basement membrane (Inset 2). The narrower bronchioles are lined by simple, cuboidal epithelium with less ciliated and more club cells (Inset 3), while the alveoli are composed of type I and type II alveolar cells (Inset 4). (**b**) Development of ciliated and goblet cell lineages. (**c**) The mucociliary escalator. (**d**) The 9 + 2 microtubular organization of a motile lung cilium. Illustration made using BioRender.com.

The respiratory system is a complex arrangement of tissues and organs whose primary function is gaseous exchange. The nostrils, the nasal cavity, and the oral cavity are the portals for entry of external air into the lungs. However, unlike the oral route, the nasal route has hair (vibrissae) for trapping unwanted air-borne contaminants. The inhaled air then journeys en route to the larynx to reach the trachea. The trachea bifurcates into the left and right bronchi on either side of the chest cavity (thorax). The bronchi enter the lungs at the hilum and further split into narrower pathways termed bronchioles. These bronchiolar branches terminate at the alveoli, the sites of large volume of gaseous exchange in our body (Figure 1a).

The respiratory system is functionally and anatomically divided into two regions. The route from the nose to the bronchioles that helps in conducting external air to the inner portions of the lungs is functionally termed the conducting zone, while the portion of the respiratory system from the alveolar duct to the alveoli is functionally termed as the respiratory zone. Based on anatomy, the human respiratory system can be divided into the upper and lower respiratory tracts (URT and LRT, respectively) (Figure 1a) [7].

The anterior portion of the nasal cavity, the vestibule, provides the portal for gaseous exchange with the external environment and is lined with stratified squamous epithelium (Figure 1a, inset 1) [8]. Subsequently, the epithelium transitions into a pseudostratified columnar type with intercalated ciliated and goblet cells (Figure 1a, inset 2) that overlie a lamina propria carrying muco-serous glands. This region also houses the basal cells, which can generate the secretory and ciliated cell lineages [5]. Approximately 60% of the cells lining the trachea are ciliated and 20% are goblet (secretory) cells, and these two directly contribute to the mucociliary function. Within the smaller airways of the LRT, the epithelium transitions to a shorter, simple, cuboidal type that is less ciliated and has a high number of club cells but reduced goblet cells (Figure 1a, inset 3) [7]. Deep within the lungs, the alveoli are composed of thin type I and type II alveolar cells that are involved in gaseous exchange and surfactant production, respectively (Figure 1a, inset 4) [9].

The goblet cells are the chief secretory cell type lining the tracheobronchial mucosa. These adhere to one another by means of tight junctions and are intercalated between the ciliated cells to form a physical barrier against external contaminants. Goblet cells are highly polarized with a basal localization of the nucleus and other organelles [7]. The apical cytoplasm is rich in membrane-bound secretory granules that contain mucin, a high molecular weight, gel-forming glycoprotein. The serous and club cells within the smaller airways have small secretory granules that produce watery secretions. The muco-serous glands in the submucosa supplement the epithelial cell secretions, and, together, they help in warming and moistening the inhaled air [10]. Together, these secretions constitute a majority of the airway surface layer.

The ciliated cells of the pseudostratified epithelium are a terminally differentiated population of epithelial cells with an elongated columnar appearance [11,12]. They are distinguished by the presence of ~300 cilia per cell on the luminal surface. The cilia have a diameter of ~0.3 µm and a length ranging from ~7 µm in the upper air tracts to ~4 µm in the narrower ones [5]. Neighboring cells are inter-connected by means of tight junctions [13]. The apical region of these ciliated cells is rich in mitochondria for sufficient ATP production that drives ciliary movement triggered by the axonemal dynein motor function [14]. The ciliated airway cells mediate the propulsion of the tracheobronchial secretion in a cephalad direction by a coordinated metachronal beating of the cilia [15].

The basal cells function as the progenitor for differentiation into the ciliated cell and goblet cell lineages. This developmental pathway begins during lung morphogenesis and is regulated by several transcription factors [16]. The inhibition of the Notch signaling pathway drives the ciliated phenotype (Figure 1b) [7]. GMNC, a coiled-coil-domain containing protein of the geminin family, has been identified to be a master regulator for ciliated cell differentiation that functions downstream of Notch signaling [17]. The transcriptional activation of GMNC turns on *MCIDAS* expression, which encodes for multicilin, another coiled-coil-domain containing protein that is a transcriptional activator of genes required for basal body production and FOXJ1 (forkhead box J1, a transcription factor), the chief controller of basal body docking, ciliogenesis, and ciliary movement [18,19,20,21]. RFX3, of the regulatory factor X family, is a transcriptional co-activator of FOXJ1 and helps to induce the expression of ciliary genes [22]. MYB, another transcription factor, has been identified to be required for ciliated cell differentiation and acts upstream of FOXJ1 [23]. Interestingly, MYB upregulation has been observed in airway diseases like COPD.

A sustained Notch activation drives secretory cell differentiation (Figure 1b) [24]. The SPDEF (SAM pointed domain-containing ETS transcription factor) has been found to be critical for pulmonary goblet cell differentiation in mice. Its overexpression, in vivo, was linked to the upregulated expression of genes like FOXA3 (forkhead box A3), AGR2 (anterior gradient 2), and GCNT3 (glucosaminyl (N-acetyl) transferase 3, mucin type) that are involved in glycosylation and goblet cell differentiation pathways [25]. SPDEF and FOXA3 overexpression has been observed during chronic pulmonary disorders such as asthma and COPD. This induces goblet cell metaplasia and mucus over-production, a key feature of several respiratory illnesses [26,27].

## 3. Structure and Composition of the MCC Apparatus in Humans

For effective MCC function, the cilia on the surface of the ciliated epithelial cells interact with the ASL. It is composed of the PCL (~7 µm) coated with an overlying layer of mucus (~2–5 µm) (Figure 1c). It also includes a thin layer of surfactant that spreads mucus all over the epithelial surface. The PCL aids in the lubrication of airway surfaces that facilitate ciliary beating [7]. In totality, the ASL is critical for normal ciliary performance and to maintain healthy airways.

Mucus is a complex, gel-like secretion of a non-static nature. It is predominantly composed of the mucin glycoproteins that provide the structural framework to the mucus. It primarily functions within healthy airways to entrap foreign pathogens and particles, dissolve toxic gases, and assist in their removal from the lungs via an effective and directional ciliary beating and cough. Thus, the mucus layer possesses a movable function, and mucus with an aberrant rheological property is a key pathological feature of chronic airway diseases. In addition, mucus acts as a reservoir of host-protective proteins and polypeptides, helps in preventing ASL dehydration, and allows for pathogen sequestration by interaction with carbohydrate ligands. The respiratory mucus contains molecules such as secretory immunoglobulin A (IgA), defensins, and histatins for host defense [28,29].

The mucins produced by the goblet cells may be secretory or membrane-associated. These molecules exist in a condensed, dehydrated state within secretory granules until their discharge, either constitutively or by external factors such as inflammatory cytokines and growth factors, bacterial components, environmental, and chemical pollutants [30]. The hyper-secretion and accumulation of mucus is a key pathological feature of diseases like cystic fibrosis, COPD, and asthma that result from dysregulated mucin production stemming from goblet cell hyperplasia. In asthmatics, an acute airway response pathway that stimulates the over-production of mucins and bronchospasm prevails, thus resulting in airway obstruction that can be fatal [31].

Mucins are composed of a mucin domain that is rich in serine and threonine residues that are O-glycosylation sites. About 70% of the mass of the mucins is contributed by the carbohydrate groups that impart to it features of superior resistance to proteolysis and the sequestration of pathogens, along with an ion and water binding function [28]. A subset of secretory mucins also contains cysteine-rich amino and carboxy termini in addition to the glycosylated mucin domain. These mucins exist in the polymeric form via the formation of intra- and inter-molecular disulfide bonds. Any alteration of the organization of mucins within the mucus layer may result in pathological diseases where the layer’s transport properties and barrier function may be compromised.

Mucins are encoded by several *MUC* genes, of which *MUC1*, *MUC3A*, *MUC3B*, *MUC4*, *MUC12*, *MUC13*, *MUC15*, *MUC16*, *MUC17*, *MUC20*, and *MUC21* code for cell surface-associated mucins, and seven genes code for the secretory type (HUGO gene nomenclature committee, https://www.genenames.org/) [32]. Among the secretory mucins, MUC2, MUC5AC, MUC5B, MUC6, and MUC19 are oligomeric and gel-forming, while MUC7 and MUC8 are non-polymeric [28]. Normal mucus is a mixture of ~97% water, ~1% salt, ~1% mucins, and ~1% of other proteins [7,33]. It predominantly contains MUC5AC and MUC5B mucins with low levels of MUC2 [34,35,36].

The hydration of the ASL allows for efficient ciliary beating. ASL hydration occurs via an active transport of ions across the ciliated epithelium. Ciliated cells express the epithelial Na^+^ channel (ENaC) at their apical membrane, which allows for Na^+^ influx and the passive transport of H_2_O/Cl^−^ across the ionic gradient and into the lumen. The regulation of the Cl^−^ export occurs via the CFTR (cystic fibrosis transmembrane conductance regulator) and the CaCC (calcium-activated chloride channel) [37].

The PCL is a gel-like layer composed of the MUC1, MUC4, and MUC16 mucins and tethered mucopolysaccharides [38,39], which create an efficient lubrication layer for ciliary beating and also restrict the entry of foreign particulates. Its hydration status is also maintained via active transmembrane ionic transport [37]. The PCL volume is critical for effective MCC, as insufficient hydration causes the collapse of the mucus layer and the entrapment of cilia within the mucus, as seen in the case of cystic fibrosis [40].

### 3.1. Functional Role of Cilia in MCC

The mucus gel, carrying the entrapped contaminants (i.e., potential lung damagers), is proximally propelled via ciliary beating, which has been shown to be regulated by a variety of factors such as progesterone, nitric oxide, and secondary messengers including cAMP, cGMP, and calcium [41,42,43,44]. Normally, cilia have two beat frequencies. The slow frequency results from its intrinsic axonemal dynein ATPase activity, while a higher frequency arises due to stimulation by specific signaling molecules. External mechanical stressors can also modulate cilia beat frequency via the stimulation of ATP release [45]. Normal cilia beat metachronically with a frequency of 12–15 Hz and propel mucus with a velocity of 4–20 mm/min [5,46]. The total volume of mucus expectorated or swallowed per day is ~30 mL [5]. The cilia come in contact with the mucus layer during the forward stroke and pass underneath it on the reverse stroke, thereby propelling it forward [40]. Ciliary beat frequency, function, and number can be altered by particulate matter, airway infections, drugs, and strenuous exercise [47,48,49]. Moreover, ageing also reduces ciliary beat frequency, with cilia cross-sections displaying micro-tubular disarrangement and single central microtubules contributing to an increased incidence of respiratory infections in the elderly [50]. Such findings indicate the critical reputation of cilia in upholding respiratory health.

### 3.2. Components of the Propeller Machinery of the MCC Apparatus

The key propeller during mucociliary clearance is the cilium. The motile cilia of the lung are capable of exerting a mechanical force with their coordinated beating to create a directional flow of respiratory fluid (i.e., mucus) within the airways [5]. In addition, the sensory bitter taste receptors present on motile cilia increase calcium ion concentration, thereby enhancing the beat frequency and imparting a chemosensory function to motile airway cilia [51].

Since cilia play a key role in the MCC apparatus, ciliogenesis is inevitable for an efficient MCC. Consequently, molecules required for ciliogenesis are also critical for an error-free functioning of the MCC apparatus. Ciliogenesis begins through the formation of centrosome-derived basal bodies that travel to the apical cell surface and dock with the membrane to nucleate the development of numerous axonemal microtubules that form the structural core of the cilium. The proteins required for ciliogenesis and ciliary maintenance are transported via the bidirectional transport system discovered in *Chlamydomonas* termed as the intraflagellar transport (IFT) machinery [52,53]. The ciliary axoneme protrudes from the plasma membrane and has a 9 + 2 organization in the motile cilia, where nine doublet microtubules are arranged in an outer circle surrounding a central core of a pair of microtubules (Figure 1d) [54,55,56]. The nine peripheral doublet microtubules possess outer and inner dynein arms that regulate microtubule sliding and ciliary motion. Outer dynein arms are present at 24 nm intervals and are critical for beat frequency, while the inner dynein arms, located at every 96 nm along the axoneme, are responsible for the wave-like formation during ciliary beating [57]. The dynein arms are linked to the central pair of microtubules via radial spokes to generate the whip-like motion that drives mucus expulsion.

### 3.3. Molecular Network of the Lung Cilia and MCC Machinery Regulating Mucociliary Clearance in Humans: A Protein Network Analysis

**Figure 2:**
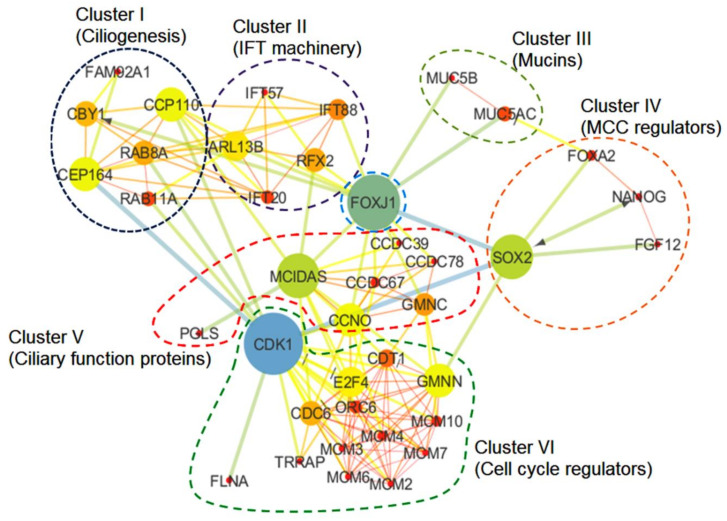
The mucociliary clearance (MCC) regulatory protein network. The principal nodes and paths that drive the mucociliary clearance regulatory protein network were analyzed using Cytoscape 3.7.2. The protein network analysis was performed by using the betweenness centrality algorithm where the sizes of the nodes (circles) indicate betweenness. The color scale ranges from blue–green–yellow to orange, which is indicative of low to high betweenness values, respectively. The clustering analysis was performed by employing the Markov clustering (MCL) algorithm. The perforated shapes represent different clusters (I–VI).

Multi-ciliated cells are involved in the control of directional fluid flow across epithelial tissues. In the conducting airways, cilia along with the secretory cells are responsible for mucociliary clearance, which expels out pathogens from the lung. The coordinated movement of the airway cilia generates a motive force that drives mucociliary clearance, which averts the infection of the respiratory tracts by pollutants and infectious microorganisms responsible for several acute and chronic diseases of the lungs. To comprehend the overall molecular mechanism regulating the mucociliary clearance, we selected 39 proteins reported to be involved either directly or indirectly in this process by referring to relevant research papers and performed an in silico protein–protein interaction analysis (detailed methodology is mentioned in the Supplementary Material, M&M S1). We included both experimentally validated and predicted protein–protein interactions in our analysis of the protein network. The network of the selected proteins was made using String version 11 (Academic Consortium 2020), and the output was further analyzed using Cytoscape 3.7.2 (San Diego, CA, USA) by employing the betweenness centrality algorithm [58,59]. The betweenness centrality algorithm determines the shortest path between each pair of nodes in a network [60]. The nodes with high betweenness value actually have the determining hold over the network. The evidence supporting the interactions between selected proteins have shown the FDR (false discovery rate) values < 0.05, which represent the significance of the association (Table S1). The clustering of the protein network was performed by employing the Markov clustering (MCL) algorithm [61]. The constructed network consists of 39 nodes that belong to different clusters (clusters I–VI) representing proteins associated with the relevant processes of mucociliary clearance system, i.e., ciliogenesis, IFT machinery, mucins and MCC regulation, ciliary functions, and cell cycle regulation (Figure 2). The betweenness analysis revealed that eight proteins (CDK1, MCIDAS, SOX2, FOXJ1, CEP164, Chibby-1 (CBY1), CCP110 and ARL13B) may behave as the principal nodes and control the whole protein network. CDK1 and FOXJ1 are the main effectors in the network and regulate many of the output nodes in the network. Additionally, the network analysis revealed three critical connections (i.e., SOX2-FOXJ1, SOX2-CDK1, and CDK1-CEP164) that might be critical for maintaining balance in the network. Clusters I and II consist of proteins involved in ciliogenesis and the IFT system (Figure 2). Cluster I has a basal body protein, Chibby (CBY), as one of the principal nodes. It is essential for accurate cilia structure and mediates the localization of the IFT machinery to the ciliated cells of the airway epithelium, the impairment of which affects mucociliary transport due to defective ciliogenesis (marked by a drastic reduction in the airway cilia number) and results in chronic upper airway infection [62,63]. The inactivation of CBY causes the accumulation of IFT88 along with other IFT-B complex proteins including IFT20 and IFT57 (part of cluster II) in the distended tips of the cilia [62]. In addition, a distal appendage protein, CEP164, is crucial for ensuring the proper recruitment of CBY and its associated proteins, FAM92A and FAM92B, to the base of the cilium in multi-ciliated cells, as well as the recruitment of IFT components to the multi-cilia [63,64]. The recruitment of CBY is an important step for the ciliary targeting of small GTPases like RAB8, RAB11, and ARL13b, in the multi-ciliated cells. The interaction of CBY with Rabin8 (a guanine nucleotide exchange factor for the small guanosine triphosphatase RAB8) promotes the recruitment of RAB8 and the efficient assembly of the ciliary vesicles [63]. Another ciliary protein, LRRC56, in conjunction with IFT88, is involved in dynein transport to the tip of the cilia. It was reported that biallelic variants of LRRC56 cause chronic respiratory infections due to dyskinetic cilia showing phenotypes like an abnormal cilia beating pattern and the absence of outer dynein arms (ODAs) in the distal region of the axoneme [65]. Cluster I also shows CCP110 as a principal node, which is a distal centriolar protein considered to be an important regulator in primary cilia assembly and motile ciliogenesis [66,67,68,69]. During ciliogenesis, the removal of CCP110 from the mother centriole is a pre-requisite for ciliation to occur [66]. The optimal level of CCP110 is regulated at the transcriptional and post-transcriptional levels in multi-ciliated cells [70]. In multi-ciliated cells, ciliation is controlled via a conserved transcriptional cascade, where the inhibition of Notch signaling activates multicilin (*MCIDAS*) followed by the establishment of a ternary complex with E2F-4/5 and Dp1 [18,71]. The ternary complex activates downstream ciliary transcription factors, including RFX2 and FOXJ1, which further regulate the expression of core ciliogenesis genes, while other cell cycle genes remain off [19,72]. Walentek and co-workers demonstrated the enhanced CCP110 expression during the inhibition of Notch signaling or the stimulation of multi-ciliogenesis [70]. E2F4, RFX2, and FOXJ1 were also reported to bind at the transcriptional start site of CCP110 [70]. Furthermore, the miR-34/449 miRNA-based post-transcriptional regulation of CCP110 was reported, which controls basal body maturation/docking and ciliogenesis in multi-ciliated cells [73]. Furthermore, Mercey and co-workers demonstrated the miR-34/449-dependent modulation of small GTPase (R-RAS) pathways to promote the assembly of the apical actin network [74]. The apical actin network assembly is considered a pre-requisite for the proper anchoring of centriole-derived neo-synthesized basal bodies during later stages of multi-ciliated cell differentiation. Walentek and co-workers demonstrated that, like CCP110, miR-34/449 expression is also activated by ciliary transcription factors [70]. Multicilin initiates the assembly of centriole in G0, an early step in multi-ciliated cell differentiation [19]. In the *MCIDAS* mutant, respiratory epithelial cells were shown to have a primary ciliary dyskinesia-like phenotype, i.e., they had one or two cilia on each cell that lacked proteins involved in ciliary motility (DNAH5 and coiled-coil domain containing protein 39 (CCDC39)) [75]. *MCIDAS* mutants also lack FOXJ1-regulating axonemal motor protein, as well as *CCNO* expression, thus suggesting that multicilin is the chief regulator of *CCNO*/FOXJ1 for human multi-ciliated cellular differentiation [75].

In the network (Figure 2), cluster V showed a cyclin O (*CCNO*) protein, which has a regulatory role in deuterosome formation and the amplification of centrioles in multi-ciliated cells [76]. Mutations in *CCNO* cause ciliary dysfunctions (RGMC, i.e., reduced generation of multiple motile cilia in respiratory epithelia) due to impaired deuterosome formation [76,77]. Deuterosomes act as amplification platforms for the generation of centrioles in multi-ciliated cells. In humans, mutations in *CCNO* are also associated with severe airway diseases. Wallmeier and co-workers executed a whole-exome sequencing approach to identify recessive *CCNO* mutations in several patients with chronic destructive lung disease as a result of insufficient airway clearance [77]. *CCNO* functions downstream of the multicilin (*MCIDAS*) and directs multi-ciliated cell development. Apart from multicilin (*MCIDAS*) and cyclin O (*CCNO*), GEMC1 (*GMNC*), a protein known for its role in the regulation of DNA replication, has also been identified to cause mucociliary clearance disorders, i.e., a reduced generation of multiple motile cilia (RGMC) in humans. Terre and co-workers demonstrated impaired growth, hydrocephaly with a high penetrance, and infertility in mice lacking GEMC1 due to defective multi-ciliated cells in the brain, respiratory tract, and germline [78]. In ciliated epithelia, GEMC1 up regulates key transcriptional regulators of multi-ciliogenesis, i.e., *MCIDAS* and FOXJ1, where GEMC1 activity is stimulated by E2F5 and inhibited by geminin [79].

In cluster VI, we observed the presence of CDK1 (cyclin-dependent kinase 1), a serine/threonine kinase that plays a crucial role in cell cycle regulation. In response to DNA damage, the cell recruits a protein complex consisting of proteins from the poly-ADP-ribose polymerase (PARP) family, MRE11, RAD40 and NBS1 to induce cell-intrinsic checkpoints. This protein complex activates the ATM/CHK2 or ATR/CHK1 pathway, both of which converge towards cell division cycle 25 (CDC25) phosphatase located upstream of CDK1/cyclin B. The CDK1 and CEP164 interaction in the network could be explained by the fact that the ATM/ATR protein kinase phosphorylates CEP164 causing the activation of CHK1, which inhibits the cyclin-A-dependent activation of CDK1 and thus pauses the progression of the cell cycle [80]. We also observed the transactivation/transformation-domain-associated protein (TRRAP; regulator acting upstream of multicilin), which binds to upstream promoter region of several genes (associated with human ciliopathies) and regulate multi-ciliated cell differentiation and function [81]. Herceg and co-workers (2001) demonstrated that TRRAP is necessary for mitotic checkpoint and normal cell cycle progression. CDK1 activity in the TRRAP mutant was compromised, which resulted in a failure of mitotic arrest [82]. Another important protein observed in cluster VI was CDC6, an ATPase that is known to be involved in the recruitment of pre-replicative complexes (pre-RC) at origins of replication during the G1 phase, as well as having a role in checkpoint activation and maintenance [83]. During pre-RC formation, CDC6 ATPase regulates the effective loading of minichromosome maintenance (MCM) proteins along with their associated factor CDT1 onto the origin of replication, whereas the origin of replication complex (ORC) ATPase causes the release of each loaded MCM unit from the ORC-CDC6 loading machine [84,85]. The proteolytic regulation of cellular CDT1 level by SCFSkp2 or Cul4-DDB1Cdt2 ubiquitin ligases is crucial for MCM loading. Moreover, the inhibitor protein geminin (GMNN) is also known to be involved in the regulation of CDT1 activity [86].

### 3.4. Physiological Importance of the MCC Apparatus and Associated Disease

**Figure 3:**
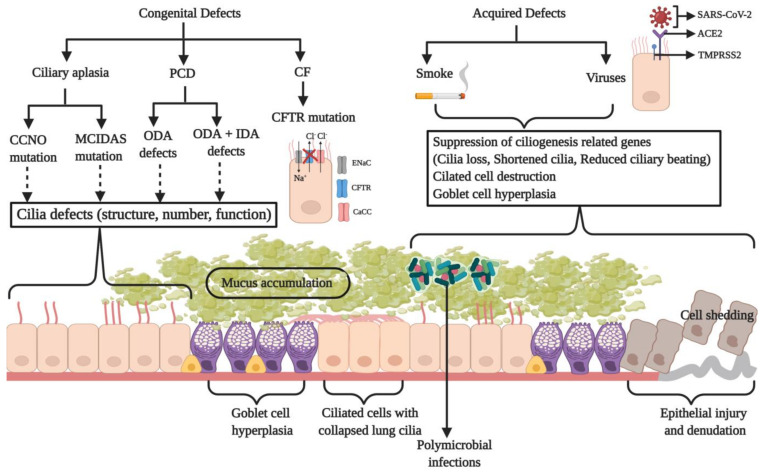
A schematic illustration of the genetic or acquired defects in the respiratory MCC apparatus that impair the clearance mechanism of the respiratory airways and lead to a diseased state. Defective cilia structure, function, or cilia loss may occur due to genetic (primary ciliary dyskinesia (PCD), ciliary aplasia), acquired (smoke), or viral factors, and they could manifest in the form of diseases like chronic obstructive pulmonary disease (COPD), asthma, and PCD where an inadequate mucus expulsion causes its build-up in the airways. During cystic fibrosis, a faulty cystic fibrosis transmembrane conductance regulator (CFTR) and goblet cell hyperplasia result in the reduction of periciliary layer (PCL) volume and mucus hyper-secretion, respectively. This causes the cilia to collapse, thereby resulting in inefficient MCC. The accumulated mucus promotes microbial colonization and the exacerbation of the disease. Microbial infections aggravate the condition and eventually damage the epithelial lining as a result of ciliated cell shedding. Illustration made using BioRender.com.

The MCC is a crucial defense mechanism against chronic airway diseases and infections. A breach in this protective barrier permits colonization and infection by pathogens. Anomalies of MCC may have a genetic basis or may be acquired defects that compromise the defense mechanism against foreign agents invading the lung (Figure 3). Prolonged smoking impairs mucociliary activity with temporary delays occurring after each smoke [87]. The bronchial epithelium of smokers displays pathological changes induced by cigarette smoke like hyperplasia, metaplasia, the presence of cells with atypical nuclei, and cilia loss [88]. Shortened airway cilia in smokers are also associated with a reduced MCC rate [89]. It was suggested that smoking induces the expression of epidermal growth factor (EGF) by the ciliated cells that bind to its receptor (EGFR) on the basal cells. This shifts the basal cell differentiation towards the squamous phenotype with the downregulation of ciliogenesis and secretory differentiation genes, as seen in the airways of the smokers [90]. The components of cigarette smoke suppress the expression of genes involved in ciliogenesis, and the overexpression of FOXJ1 was found to reverse this effect in vitro [91]. Such anomalies in the MCC apparatus such as a patchy or generalized loss of cilia, squamous metaplasia, and the hyperplasia of goblet cells are also found in pediatric patients exposed to passive smoking [92]. These changes in the nasal mucosa ultrastructure may affect mucociliary function, and such kids may develop persistent sinus infections with increased severity associated with prolonged exposure to smoke chemicals. The shortening and loss of cilia also results from the use of drugs like marijuana and cocaine, as well as a range of environmental pollutants, thereby posing a significant health risk by impairing MCC [5,93,94].

COPD is a progressive, chronic, inflammatory lung ailment characterized by emphysema and chronic bronchitis that blocks the airflow through the respiratory passage, making it difficult for the patient to breathe and leads to wheezing. It occurs due to long term exposure to chemical irritants, predominantly tobacco smoke. Such gaseous particles inflame the bronchial mucosa causing chronic bronchitis characterized by the frequent production of cough and mucus. The alveoli are damaged as a result of prolonged exposure to chemical irritants, thus causing emphysema. COPD patients show even smaller lung cilia than the healthy smokers and a sluggish ciliary beating consistent with the presentation of an impaired MCC [49,95].

Asthma is a major non-communicable chronic condition resulting from the inflammation and constriction of the respiratory tract due to allergen exposure. Patients may experience wheezing, coughing, and tightness in the chest during an asthma attack. Dunnill reported the presence of mucus plugs in the bronchial tracts, the shedding of the ciliated cells of the bronchial mucosa, and the impaired clearance of bronchial secretion during autopsy of patients with asthma [96]. Histopathological changes observed in the bronchial biopsy specimens of asthmatic children and adults revealed damage to the epithelial layer with the loss of cilia, the degranulation of mast cells, and the destruction of ciliated cells [97,98]. The ciliary ultrastructure showed ciliary defects and dysfunction (dyskinetic and immotile cilia) with a reduced beating frequency, especially in the severely affected patients with no significant alterations in cilia length [99]. The Th2 cytokine, interleukin-13 (IL-13), is regarded as a key effector molecule for goblet cell metaplasia, reduction of cilia beat frequency, eosinophil infiltration, IgE production, mucus hypersecretion, and bronchial hyper reactivity associated with asthma [100,101,102]. Human airway epithelial cells cultured in the presence of IL-13 showed a loss of ciliated cells due to reduced FOXJ1 expression manifesting in basal body and ezrin mis-localization [103]. Polymorphisms in the kinesin family member, *KIF3A*, have been identified as novel candidates for childhood asthma [104].

A rare congenital disorder of the mucociliary apparatus termed as “ciliary aplasia” results in a drastic reduction in the number of motile cilia in the respiratory epithelium [105]. Patients exhibit symptoms of classical PCD such as recurrent pulmonary infections (along with rhinitis, cough, sputum production, wheezing, fever, and nasal discharge) and infertility [106]. In 2014, a research group adopted a whole-exome genome sequencing strategy and identified that the loss-of-function or missense mutations in the *CCNO* and *MCIDAS* genes (coding for cyclin O and multicilin, respectively) are associated with the “reduced generation of multiple motile cilia (RGMC),” thus leading to a rare mucociliary clearance disorder in the airway epithelium [75,77]. During ciliogenesis, cyclin O is essential for the production of basal bodies. Consistent with its predicted mode of action, in vitro ciliogenesis experiments have shown the defective generation and localization of centrioles in *CCNO* mutant cells. *MCIDAS* lies adjacent to *CCNO* on the 5q11 chromosome and is also a key player in ciliated cell differentiation [18]. Both function in the same pathway upstream of FOXJ1, and multicilin regulates the expression of both these proteins. Both *CCNO* and *MCIDAS* mutations were found to be consistent with an autosomal-recessive inheritance pattern and clinically manifested as postnatal respiratory distress and recurrent chronic infections of the URT and LRT including nasal polyps and sinusitis, rhinitis, otitis media, bronchiectasis, chronic obstructive airway disease, recurrent pneumonia, and infertility. A chest CT (computerized tomography) scan showed chronic destructive lung disease with bronchiectasis and mucus plugging. The magnetic resonance imaging (MRI) of the brain revealed hydrocephalus in a few patients. Respiratory insufficiency and failure necessitated lung transplantation and was also associated with mortality in a few cases. The TEM of respiratory epithelial cells showed lesser number of cilia along with basal body mis-localization. Mutations in *CCNO* showed some residual ciliary motility, while it was completely absent in *MCIDAS* mutations.

Another congenital disorder that impairs mucociliary clearance is cystic fibrosis. It is an autosomal recessive genetic disorder resulting from mutations in the *CFTR/ABCC7* gene that encodes for an ABC (ATP-binding cassette) transporter [107]. CFTR functions as an ATP- and cAMP-dependent Cl^−^ ion channel situated at the apical membrane of the cells lining the respiratory epithelium [108]. Several mutations have been identified to be associated with the hyper-secretory disease, but Phe508 deletion is the most common [109,110]. A dysfunctional CFTR within the epithelial cells results in a reduced secretion of Cl^−^ ions, the dehydration and depletion of the ASL, an increased Na^+^ absorption, and a 5–10 fold higher mucin to water ratio with an enhanced viscoelasticity [111,112]. This causes the mucus layer above to attach to the ciliated cells below, thereby impairing ciliary beating and mucociliary clearance (Figure 3). As a result, a thick layer of mucus develops within the air tracts, thus providing a niche for microbial colonization (Figure 3) [113]. Microbes trigger episodes of acute inflammation and pulmonary exacerbation responsible for the deterioration of lung function. The sputum microstructure is significantly altered by elevated mucin and extracellular DNA content. A high sialylation of MUC2 and MUC5AC was detected in the patients suffering from CF [114]. These mucins express highly sialylated and sulphated Lewis x determinants, which are attachment sites for *Pseudomonas aeruginosa*, the microorganism most responsible for morbidity and mortality associated with CF. These results indicate a co-induction of the expression of mucin genes along with glycosyl- and sulfo-transferases during airway inflammation. To make matters worse, in neonates with a *CFTR* mutation, the ASL has an acidic pH due to faulty bicarbonate secretion, which reduces its antimicrobial activity [115,116]. These factors contribute to the persistence of infection and inflammation, thus causing a decline in lung function.

### 3.5. Ciliopathies and Their Relevance to MCC

Since motile cilia are crucial in establishing mucus flow and clearance within the respiratory tract, any violation of their orchestrated movement promotes the development of respiratory disorders due to an ineffective MCC. Approximately 5% of the children suffering from chronic respiratory infections are diagnosed with a ciliopathy known as primary ciliary dyskinesia (PCD), which is predominantly an autosomal recessive genetic trait, although it has also been rarely reported to be autosomal dominant or X-linked [117]. It is a heterogenic disorder of the motile cilia occurring in 1 in 15,000 live births and arises due to mutations in several genes responsible for cilia structure, function, and assembly or biogenesis [7,118]. Most of these defects can be grouped under (a) short or absent outer dynein arms (ODA) and (b) both outer and inner dynein arm (ODA and IDA, respectively) defects. Some of the common clinical presentations of the disease include neonatal respiratory distress (in 80% of cases) and recurrent pneumonia, chronic rhinitis, nasal congestion and bronchiectasis, organ laterality defects, chronic sinusitis, chronic otitis media, and infertility [119,120,121]. It is also associated with a significant reduction in cilia beating frequency, abnormal ciliary wave form, and the absence of mucociliary transport (Figure 3) [122]. Patients exhibit a retarded airway clearance that allows a longer residence time for pathogens, thereby resulting in recurrent LRT infections that lead to bronchiectasis and lung transplantation in the severe conditions [123].

The mutations in the genes, *DNAI1* (axonemal dynein intermediate-chain gene 1) and *DNAH5* (dynein axonemal heavy chain 5), encoding the components of the ODA manifest in the form of immotile or hypokinetic cilia. These were the first genes identified to be associated with PCD [124,125]. A homozygous mutation of the *DNAL1* gene encoding the outer dynein arm light chain 1 via the alteration of the Asn150 reduced the stability of the axonemal dynein light chain 1 and was linked to PCD [126]. Other PCD-associated mutations that cause ultrastructural defects in the outer dynein arm were observed in *DNAI2* and *TXNDC3* (thioredoxin-nucleoside diphosphate kinase) [127,128]. *DNAH11* mutations, however, showed a hyperkinetic ciliary beating, albeit a normal axonemal ultrastructure, and were thus identified by genetic testing [129,130]. ODA defects lower or completely eliminate cilia beating frequency [131]. Other mutations in the ODA docking complex proteins are those in *CCDC151* and *CCDC114* that result in outer dynein arm defects that cause ciliary dysmotility and severely reduced ciliary beating [132,133]. Proteins involved in the assembly of the IDA and the dynein regulatory complex, CCDC39 and CCDC40, were also mutated during PCD [134,135]. CCDC 40 is required for the axonemal recruitment of the former and is vital for ciliary motility and the formation of the left–right axis [135]. *HYDIN* and *RSPH4A* are genes that encode the central apparatus and radial spokes, while RSPH1 and RSPH9 are radial spoke head proteins. Their mutations caused motility defects and resulted in absence of the central pair of microtubules, resulting in a rotational motion of cilia [136,137,138,139]. A normal beat frequency, albeit an abnormal circular motion beat pattern, was associated with milder clinical diseases (with features such as better nasal nitric oxide levels, reduced incidences of neonatal respiratory distress, delayed commencement of cough with sputum, and better lung function) in *RSPH1* homozygous mutations [139].

In PCD, disease variation and severity are associated with mutations in specific genes. The biallelic mutations causing a loss of function of *CCDC39* and *CCDC40* result in axonemal disorganization, as noted by the absence of inner dynein arms and disordered microtubules in some cilia. It has been reported that these mutations were associated with severe lung disease in children [117,140]. The CCDC 39 and 40 complex acts as a molecular ruler to construct the accurate 96 nm spacing of the doublet microtubules in the ciliary axoneme [141]. *CCDC65* mutations have a normal axonemal ultrastructure with hyperkinetic cilia [142]. Several PCD-associated mutations have been found by comparative genomics and encode cytoplasmic proteins involved in cilia assembly and protein trafficking. Such mutations cause ultrastructural abnormalities such as truncated or missing IDAs and ODAs, thus resulting in immotile cilia or severely impaired ciliary beating. These include mutations in *HEATR2* [143], *ZMYND10* [144], *SPAG1* [145], *LRRC6* [146], *ARMC4* [147], DNAAF1 [148], *DNAAF2* [149], *DNAAF3* [150], *CCDC103* [151], and *DYX1C1* [152]. Recently, a mutation in the *CFAP57* gene has been identified to be associated with PCD. This protein is involved in IDA assembly, and its absence resulted in a reduced beating frequency of cilia with an altered beating pattern [153].

Chivukula and co-workers recently showed the correlation of a loss of function mutation in the *NEK10* (NIMA-related kinase 10) gene to the development of familial bronchiectasis in an autosomal recessive manner [154]. In the human airways, *NEK10* specifically expresses in a ciliated cell-specific fashion and is required for an effective MCC. Its deficiency was seen to reduce the ciliary length. NEK10 was shown to affect diverse components of the ciliary proteome such as axonemal dynein and assembly factors, kinesins, proteins of the IFT machinery, and proteins regulating ciliary length. Studies in *Chlamydomonas reinhardtii* determined that cilium length was critical to ciliary beating, and shorter cilia failed to achieve periodic beating [155]. Thus, NEK10 is essential for generating ciliary movement in the airway for mucociliary clearance.

### 3.6. Mucociliary Dysfunctions upon Polymicrobial Infections

The air tracts of patients suffering from respiratory diseases can be chronically inhabited by complex communities of diverse microbes [156,157,158]. Several microbial pathogens like *Bordetella*, *P. aeruginosa*, *Mycoplasma pneumoniae*, and *Actinobacillus pleuropneumoniae* selectively target and bind to the ciliated cells [159]. These polymicrobial infections are specific to an individual and are important factors in determining the host–pathogen interaction, altering the lung environment apart from influencing the disease progression, the course of treatment, and the clinical outcome of the disease. *P. aeruginosa* is the commonly isolated microbe from the sputum samples of adult patients with PCD, while *Haemophilus influenzae* colonization is prevalent till adolescence [160,161]. However, other bacterial pathogens, such as *Staphylococcus aureus, Streptococcus pneumoniae, Ralstonia, Moraxella catarrhalis*, nontuberculous *Mycobacteria*, and *Achromobacter xylosoxidans*, have also been identified in PCD sputum [162,163,164].

In patients with cystic fibrosis, polymicrobial infections trigger pulmonary exacerbations causing irreversible lung damage and increase the incidence of morbidity and mortality accompanying the disease [165,166]. An accelerated worsening of disease symptoms such as shortness of breath and increase in respiratory rate, weight loss and appetite loss, increased coughing and sputum production, hemoptysis (coughing up blood), reduced lung function, and an increased neutrophil count are key characteristics of pulmonary exacerbation [167]. While antimicrobial therapy constitutes an effective component of pulmonary exacerbation treatment, over time, the lung function declines and requires transplantation [168,169]. Children with cystic fibrosis have been commonly found to carry *H. influenzae* and *S. aureus* infections within their airways, while the microbial community changes to *P. aeruginosa* or *Burkholderia cepacia* in adults [170]. The neutrophil elastase and proteinases produced by *P. aeruginosa* are cytotoxic, reduce ciliary beat frequency, and cause epithelial damage, thus contributing to a delayed MCC [171,172]. Furthermore, the reactive oxygen species produced by the polymorphonuclear leukocytes in response to infection decrease the beating frequency of respiratory cilia [173]. A sputum analysis from 14 adult CF patients showed the presence of seven core genera belonging to both aerobic as well as anaerobic bacteria, namely, *Pseudomonas*, *Streptococcus*, *Neisseria*, *Catonella*, *Porphyromonas*, *Prevotella*, and *Veillonella* [174]. The *Streptococcus milleri* group (SMG) was identified as the cause of chronic pulmonary infections [175]. The fungal *Candida* spp., *Malassezia* spp., and *Aspergillus fumigatus* were determined to be predominant in the mycobiome of the sputum from CF patients, while influenza A/H1N1 and respiratory syncytial virus were prevalent in throat swabs of CF patients presenting with acute pulmonary exacerbation [157,158,169]. The aggressive antimicrobial treatment of such conditions has led to the emergence of multiple drug-resistant (MDR) non-tuberculous mycobacteria, the *Burkholderia cepacia* group, methicillin-resistant *S. aureus* (MRSA), vancomycin intermediate *S. aureus* (VISA), and *Trichosporon* spp. as infectious agents in CF patients. In addition, advancements in molecular profiling have helped in identifying other microbial contributors responsible for chronic lung infections in CF such as *Streptococcus anginosus* and rhinovirus [169].

Apart from a genetic or acquired environmental cause, ciliary disorientation and mucociliary dysfunction may also stem from inflammation as a result of microbial infections [176,177]. Pulmonary infections like bacterial pneumonia and COPD have often been observed in the HIV-infected population [178]. It was demonstrated that the bronchial epithelium can be infected with HIV because it expresses the HIV receptors and co-receptors such as CD4, CCR5 and CXCR4 [179]. This infection interfered with epithelial cell differentiation and suppressed ciliogenesis. In addition, the TAT protein of the virus was found to suppress CFTR biogenesis via a TGF-β signaling pathway. HIV-infected patients often exhibit recurrent sinus infections due to a delayed MCC. The nasal nitric oxide inhibits bacterial and viral growth in the upper airways and has been reported to be about 21% lower in HIV-infected individuals thereby contributing to an increased susceptibility to airway infections [180]. The MTT (mucociliary transport time) in non-HIV control individuals was found to be 7.4 ± 3.7 min as opposed to 11.9 ± 5.9 min in patients infected with HIV. Disease progression from HIV to AIDS, as well as a history of sinus infections, further delayed the clearance rates to 13.5 ± 6.8 and 13.7 ± 6.8 min, respectively [181].

The respiratory viruses are a major cause of infections that can inflame and injure the human airways. Such viral infections also stimulate the development of secondary bacterial infections as a result of the impaired integrity of the mucociliary epithelium [182,183]. Additionally, in individuals with existing airway diseases, viral infections may exacerbate the existing medical symptoms and may be fatal [184,185]. A predisposition to superinfections by *H. influenza*, *S. aureus*, and *S. pneumoniae* is well-documented for airways infected with influenza or respiratory syncytial virus (RSV) [182,183]. Researchers compared the transcriptomic signatures of respiratory viral infection by influenza, hRSV (human respiratory syncytial virus), and hMPV (human metapneumovirus) [186]. In HAE (human reconstituted airway epithelial) models, the influenza viral peak was attained earlier than hMPV or hRSV, which corroborated well with changes in the epithelial surface observed via microscopy. There was significant induction of the pro-inflammatory Th1 response cytokines IL-2 and IP-10 (interferon gamma-induced protein 10), while RANTES (regulated on activation, normal T cell expressed and secreted), IL-8, IL-6, MIP-1B (macrophage inflammatory protein-1B), IL-1B, and IL-1RA were induced to a lesser extent. Notably, hMPV infection did not show a relative increase in RANTES and IL-8, while hRSV did not show a significant effect on GM-CSF (granulocyte-macrophage colony-stimulating factor). In all cases, there was a significant downregulation of the ciliogenesis-related genes. Mucociliary movement was completely eliminated in the case of hRSV infection but was only reduced in the case of hMPV. The MCC rate was reduced more than two folds in the case of influenza virus infection in comparison to mock.

## 4. Coronavirus Disease Manifestation in the Respiratory System

The viruses of the order Nidovirales are enveloped and possess large, positive sense, single stranded RNA genomes that are non-segmented and can range up to 26–32 kb [187,188]. Within this order lies the family Coronaviridae and the subfamily Coronavirinae, which houses the four genera of coronaviruses viz alpha, beta, gamma, and delta [189]. The coronaviruses (CoVs) are known to infect both animals and humans. Of extreme relevance to this review are the etiological agents for respiratory tract illnesses in humans.

Among the first described coronavirus strains associated with human infections are HCoV-229E (alphacoronavirus) and HCoV-OC43 (betacoronavirus) that cause mild URT illnesses in man. Indeed, along with the later discovered HCoV-NL63 (alphacoronavirus) and HCoV-HKU1 (betacoronavirus), these viruses are endemic in humans and contribute to 15–30% of the common colds annually [187] that progress to severe LRT infections in the case of infants, elderly, and the immunocompromised [189]. However, the occurrence of the severe acute respiratory syndrome (SARS) during 2002–2003 changed the picture. SARS was the first epidemic of the 21st century caused by an emerging virus from cross-species viral transmission (zoonosis) and subsequent human to human transmissions. Originating in Southern China as an outbreak of pneumonia, the disease soon spread across several countries and has resulted in 8000 confirmed cases with a mortality of 9.6% [190,191]. SARS-CoV, a betacoronavirus, which possibly originated in bats, was identified as the etiological agent of SARS [192]. The disease varies in its severity from asymptomatic to mild influenza-like symptoms, and in extreme cases, the onset of a severe form of disease causes acute respiratory distress syndrome, respiratory failure, and death. Age and co-morbid illnesses are key factors that enhance the severity of the disease [193].

The clinical course of SARS follows three phases, namely viral replication, immune hyper-reactivity, and pulmonary tissue deterioration [194]. During the first phase of infection, patients develop fever with an increase in the viral load in bodily secretions such as stool, urine, and respiratory secretions. There is a gradual increase in lung damage as evident from X-ray and CT scans [195]. Phase 2 is marked by the recurrence of fever, reduction in viral load, oxygen desaturation (SPO_2_), the development of pneumonia, and respiratory distress syndrome. In the third phase, a sustained pulmonary damage with a honeycomb-like appearance of lungs on the CT scan, lung fibrosis, and a lack of oxygen in blood necessitates the intensive care and ventilation of the patients. Finally, the patient might enter a fatal stage. Recovered patients show signs of long-lasting pulmonary damage [196,197,198].

The most commonly reported symptoms of SARS include fever, dry cough, chills, myalgia, and malaise [194,199]. Patients report a shortness of breath and a lung CT scan shows consolidation by the end of the first week. Disease manifestations in other organs include diarrhea, renal and liver dysfunctions, thrombocytopenia, lymphopenia with a decrease in CD4^+^ and CD8^+^ T cells with thrombo-embolic events, and neuro-invasion occurring in a few cases [200,201,202,203,204,205,206,207]. There is the activation of Th1 cell-mediated immunity and inflammatory response characterized by a marked elevation of interferon gamma (IFN-γ); IL-1, 6 and 12; the neutrophil chemokine IL-8; monocyte chemoattractant protein-1 (MCP-1); and the Th1 chemokine IP-10 [208].

In 2012, the Middle East respiratory syndrome (MERS) caused by the MERS-CoV (betacoronavirus) was the sixth case of coronavirus to cause human infections, yet again via an interspecies jump from bats to humans with dromedary camels as an intermediate host [209,210,211]. Globally, there are more than 2000 confirmed cases of MERS with a mortality ratio of 34.4% [212]. Like in the case of SARS, a MERS-CoV infection may vary from mild symptoms to severe acute respiratory disease and death, especially in the elderly and patients with underlying co-morbidities like diabetes, hypertension, and renal and cardiac disease [213,214,215].

The year 2019 ended in the grip of a previously unknown novel coronavirus termed SARS-CoV-2 or 2019-nCoV (betacoronavirus), the causative agent of the COVID-19 pandemic. Originating in the Wuhan city of China as ‘a pneumonia of unknown origin,’ this emerging virus has affected the lives of millions of people globally and has become an enormous threat to the public health and global economy. SARS-CoV-2 constitutes the seventh member of coronaviruses known to infect humans. The genomic characterization of the virus indicated a genome of 29.8 kb with similarity to corona virus of bat origin [216,217]. It was found to be similar to SARS-CoV in terms of its transmission and pathogenicity, and it uses the receptor ACE2 (angiotensin converting enzyme 2) and TMPRSS2 protease (a type II transmembrane serine protease) for cell entry (Figure 3) [218]. Single-cell RNA sequencing data showed that these elements that mediate SARS-CoV-2’s entry into the body are predominantly expressed in the nasal epithelial cells, thus providing a molecular basis for early stage viral transmission [219,220]. Under the TEM, the viruses showed little pleomorphism and were mostly spherical with 60–140 nm diameter and spikes of 9–12 nm in length [221]. The novel coronavirus possesses 16 non-structural proteins (NSPs) and four structural proteins (S: spike; E: envelope; M: membrane; and N: nucleocapsid) [222]. The S glycoprotein is involved in host cell invasion and infection. The receptor binding domain (RBD) of the S protein recognizes and attaches to the ACE2 receptor. The S protein is then cleaved by TMPRSS2 into two subunits (S1 and S2). S1 is responsible for receptor binding, and S2 is essential for fusion with the host membrane and cell entry [189]. Both ACE2 and TMPRSS2 are expressed in the secretory and ciliated cells of the airway epithelium [219,220]. ACE2 is highly expressed in the nasal epithelial cells (URT) and is significantly lower in the bronchi and lung parenchyma (LRT) [220,223]. Considering the high rate of infectivity of SARS-CoV-2, alternate mechanisms of cellular entry have recently been deciphered for this virus, particularly in tissues with a low or absent ACE2 expression. Contrary to SARS-CoV, the S glycoprotein of SARS-CoV-2 possesses a polybasic sequence at the S1–S2 junction that can be cleaved by the cellular protease furin [224]. This cleavage reaction generates the sequence ‘RRAR’ at the S1 subunit’s C-terminus that conforms to the C-end rule and can bind to neuropilin receptors on the target cells [225]. Neuropilin-1 (NRP1) has been reported to be a host factor for SARS-CoV-2 entry and potentiates its infectivity [226,227]. Interestingly, the gene expression of NRP1 and 2 was observed to be upregulated in the lung tissues of patients who died from COVID-19 [228]. The members of the C-type lectin superfamily, CD209L (L-SIGN) and CD209 (DC-SIGN), can also mediate coronavirus infection by interacting with the RBD of the S glycoprotein [229]. CD209L is prominently expressed in the epithelial and endothelial cells of the lungs and kidneys. It also interacts with the ACE2 receptor, indicating their heterodimerization and a co-receptor function to facilitate viral entry in tissues where both ACE2 and CD209L are co-expressed. CD209L has two sites for N-glycosylation (Asn92 and Asn361), of which only the former is glycosylated. The removal of the bulky N-linked glycans from this site resulted in the enhancement of the CD209L-S protein interaction. Additionally, docking studies have revealed that the RBD of the S protein houses a site for heparin/heparan sulfate binding that lies adjacent to the ACE2 binding site [230]. Based on in vitro experiments, Claussen and co-workers proposed a model of the heparan sulfate-mediated augmentation of SARS-CoV-2 binding to the ACE2 receptor [230]. Yet another novel mode of host entry and invasion by spike protein exploiting the CD147 receptor was recently demonstrated [231].

SARS-CoV-2 is less pathogenic than SARS-CoV and MERS-CoV but has shown a wider spread, thus posing challenges for its mitigation [232]. Fever, cough, and fatigue are the most commonly reported symptoms for COVID-19; however, sore throat, diarrhea, headache, sputum production, and shortness of breath have also been reported [233,234]. Patients may exhibit no symptoms or mild to severe symptoms that can be fatal. Clinical studies have shown that individuals with endocrinopathies (diabetes, hypertension, obesity, and cardiovascular diseases) show increased complications [235] associated with poor prognosis. Patients suffering from a severe disease also exhibit coagulation disorders, as observed during SARS-CoV and MERS-CoV infections with high circulatory levels of D-dimer, fibrin degradation products, and thromboembolic complications [236,237,238,239]. Thrombocytopenia, lymphocytopenia, leukopenia, and elevated C-reactive protein levels have also been observed [240]. Chemosensory dysfunction, such as smell and taste impairment, has also been accepted to be associated with COVID-19 infection among clinicians and healthcare workers [241,242,243].

### 4.1. Coronavirus-Induced Dysfunctions of the Lung Cilia

In 1994, Afzelius described the ultrastructure of the nasal epithelium upon coronavirus infection using electron microscopy, wherein virion particles showed a specific affinity towards the ciliated cells but not the goblet cells [244]. In addition, while viral infection did not induce the destruction of the ciliated cells, however the retraction of the cilia into the cell body was observed in a few cells, thus leading to ciliary loss. Such a selective attachment to ciliated epithelial cells has also been seen in case of other respiratory viruses like myxoviruses, respiratory syncytial viruses, influenza, and rhinoviruses [245,246]. The loss of cilia along with diffused alveolar damage, hemophagocytosis, bronchial epithelial denudation, and squamous metaplasia are key morphological changes that have been observed in the lungs of deceased patients from SARS [247,248]. Secondary bacterial or fungal infections were also reported in case of a few autopsies [249].

In a quest to understand the magnitude of coronavirus infection on the destruction of ciliated respiratory epithelium of the human nose, scientists inoculated 15 healthy adult volunteers with HCoV-229E via the nasal route. Eight of the 11 exposed to the active virus developed mild symptoms of URT infection like cold, cough, fever, and headache. Samples of ciliated epithelium obtained from all 11 test subjects at zero and three days after infection showed a defined loss of respiratory epithelium integrity marked by a significant reduction in ciliated cells and an increase in dead cell population. Furthermore, there was an increase in micro tubular abnormalities on the third day post-infection with ciliary dyskinesia [250]. Such a massive ciliary loss during coronavirus infections may lead to rhinorrhoea [244,251].

The etiological agent of the COVID-19 infection, SARS-CoV-2, has also been linked to ciliary loss and damage to the pulmonary epithelium (Figure 3). The bronchoalveolar-lavage fluid specimens obtained from three patients known to frequent the seafood wholesale market in Wuhan, the epicenter of the COVID-19 outbreak, were used to inoculate human airway epithelial cells cultured such that they resembled the pseudostratified airway epithelium of the respiratory system. After 96 h of inoculation, the cells exhibited cytopathy and a lack of ciliary beating under a light microscope [221].

All these morphological evidences outline the damage to the primary defense barrier of the human respiratory system, the cilia, during coronavirus infections. Ciliary loss is associated with inefficient mucus and pathogen clearance from the airways. Mucus build-up will attract secondary microbial infections that can complicate recovery from disease. Hence, MCC defects like asthma, COPD, PCD, and cystic fibrosis are risk factors for developing COVID-19. Furthermore, ciliary beat frequency is known to slow down with age [252], suggesting that the current pandemic is a real hazard for the lives of the elderly population.

Another key symptom pointing towards ciliary damage during SARS-CoV-2 infection is anosmia (the loss of the sense of smell) [253,254,255]. In the human body, such olfactory functions are linked to the sensory olfactory cilia that detect airborne stimuli and transmit electrical signals to the olfactory cortex within the brain [256]. Anosmia resulting from a deficit of proper ciliary structure or function has been observed in a ciliopathy termed Bardet–Biedl syndrome (BBS) [257]. Hypomorphic mutations of the CEP290 protein causes Leber congenital amaurosis that manifests as retinal dystrophy and severely aberrant olfactory function [258]. The interaction of SARS-CoV-2 Nsp 13 (non-structural protein-13) with centrosomal components may provide a molecular link to COVID-19 infection and anosmia [222,259].

### 4.2. SARS-CoV-2-Manifested Implications on the Molecular Machinery Regulating Mucociliary Clearance

**Figure 4:**
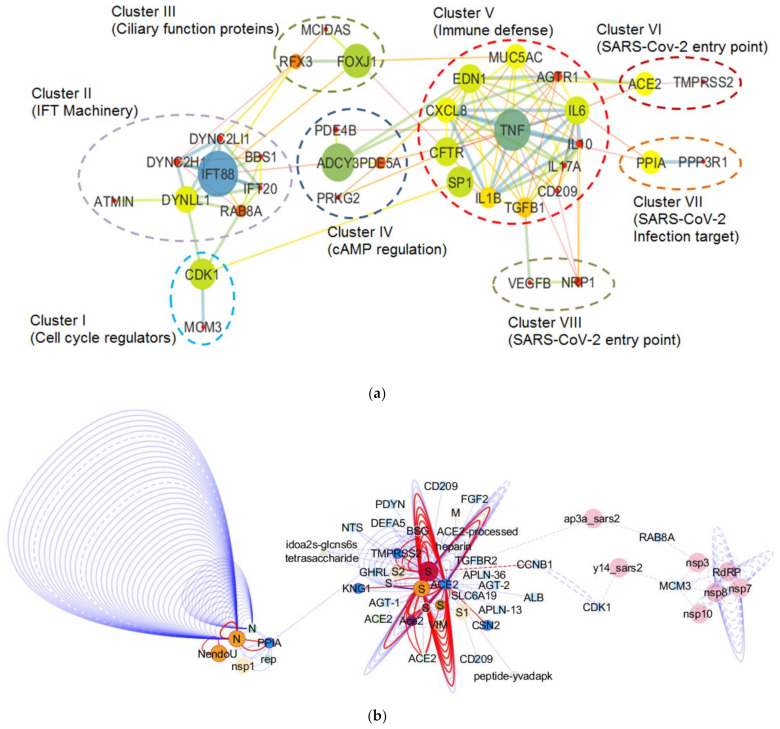
Severe acute respiratory syndrome coronavirus-2 (SARS-CoV-2)-induced changes in the molecular machinery regulating mucociliary clearance. (**a**) Interactome representing the SARS-CoV-2 infection-induced implications in the molecular machinery regulating mucociliary clearance. The principal nodes and paths driving the molecular network were analyzed using Cytoscape 3.7.2. The network analysis was performed using the betweenness centrality algorithm, where the sizes of the nodes (circles) indicate betweenness. The color scale ranges from blue–green–yellow to orange, representing low to high betweenness values, respectively. Clustering analysis was performed by employing the MCL algorithm. Perforated shapes represent different clusters (I–VIII). Clusters I–V consist of proteins involved in cell cycle regulation, IFT machinery, cilia biogenesis and regulation, cAMP regulation, and immune defense, respectively. Clusters VI–VIII represent proteins acting as SARS-CoV-2 entry point and infection targets, respectively. CDK1 (cluster I), minichromosome maintenance 3 (MCM3; cluster I), RAB8A (cluster II), CD209 (cluster V), angiotensin converting enzyme (ACE2; cluster VI), PPIA (cluster VII), and neuropilin-1 (NRP1; cluster VIII) proteins are known to interact with viral proteins during the course of infection. (**b**) Partial interactome of SARS-CoV-2 and host proteins extracted from Network Data Exchange (NDEx) public server (www.ndexbio.org) representing the IntAct/IMEx Coronavirus Dataset. The protein–protein interaction networks (PPIs) were visualized in Cytoscape 3.7.2 (with CyNDEx-2 application) with the Compound Spring Embedder (CoSE) layout. Each edge represents interaction studied in different experiments. Edge highlighted in red represents evidence from mutation study. Dashed lines represent spoke-expanded interactions. Node color shows different species origin, where pink and orange colors represent SARS-CoV-2 origin and the blue color is used to denote human origin proteins.

As discussed in the previous sections, mucociliary clearance dysfunction leads to compromised respiratory health. Considering the harmful impact of SARS-CoV-2 on the respiratory system, we aimed to comprehend the implications of SARS-CoV-2 infection on the molecular machinery regulating mucociliary clearance. To get a clear picture of the cellular players involved and the complex interactions that regulate the mucociliary clearance following SARS-CoV-2 infection, we selected 36 proteins by referring to relevant research papers and constructed a protein–protein interaction network (detailed methodology is present in the Supplementary Material, M&M S1). The FDR values representing the confidence and significance of the protein interactions are mentioned in Table S2. The obtained network consisted of 36 nodes that belong to different clusters (clusters I–VIII) representing proteins associated to the mucociliary clearance system relevant processes (Figure 4a). Clusters I–V consist of proteins involved in cell cycle regulation, IFT machinery, cilia biogenesis and regulation, cAMP regulation, and immune defense, respectively, whereas clusters VI–VIII represent proteins that act as SARS-CoV-2 entry point and infection targets, respectively (Figure 4a). The betweenness analysis revealed that seven proteins (CDK1, IFT88, FOXJ1, ADCY3, TNF, PPIA, and ACE2) may behave as the principal nodes and control the whole protein network. IFT88 and TNF are the main effectors and regulate many output nodes in the network. Cluster I shows an interaction between two cell cycle regulator proteins, CDK1 and MCM3 (Figure 4a), which are also described as components of the molecular machinery regulating mucociliary clearance (Figure 2). CDK1 phosphorylates Ser112 in MCM3, which results in its incorporation into MCM2-7 complex and loading of the MCM3 onto chromatin in cycling cells [260]. The MCM2-7 complex (ATP-dependent helicase) causes the unwinding of DNA and regulates the initiation of DNA replication as well as replication fork progression in association with ORC and Cdc6 [261]. Furthermore, CDK1 also showed interactions with DYNLL1 (a component of the dynein light chain) and RAB8A in cluster II (IFT machinery) (Figure 4a). In yeast, CDK1 controls the fidelity of chromosome segregation by regulating nucleolus spindle kinetochore 1 and dynein light chain 1 complex (Nsk1–Dlc1) at the kinetochore-microtubule interface [262]. Reports have also demonstrated DYNLL1 interactions with astrin–kinastrin/SKAP (small kinetochore-associated protein) and human NSK1-like proteins involved in controlling proper chromosome movements [263,264]. CDK1 and RAB8A separately interact with pericentriolar material 1 (PCM1) to control the assembly and disassembly of the cilia [265].

In cluster II, ATM-interactor (ATMIN) is a transcriptional regulator of the dynein light chain LC8-type 1 (DYNLL1) essential for normal lung morphogenesis and ciliogenesis [266,267]. The inactivation of the ATMIN transcriptional regulator showed a moderate but significant reduction in the expression of a ciliogenic transcriptional regulator FOXJ1 and some IFT protein-encoding loci, i.e., IFT40 (IFTA), IFT88, and IFT72 (IFTB) [266]. Goggolidou and co-workers also demonstrated the presence of bulges at the cilia base in both DYNLL1 and DYNC2H1 (component of mammalian dynein 2) mutants, which is a characteristic phenotype representing defective cytoplasmic dynein 2 functions [266]. The ATMIN mutation also affected the hedgehog signaling [266], which needs normal cilia to function in lungs [268]. It has already been reported that the lungs with defective hedgehog signaling could lead to pulmonary defects [269,270]. Moreover, impaired retrograde IFT due to mutations in the cytoplasmic dynein 2 heavy chain, DYNC2H1, and the IFT-A genes IFT122, WDR35/IFT121, and IFT43 have also been reported to cause lung mis-patterning [271].

Cluster III shows an interaction between MCIDAS and FOXJ1, two of the important factors that regulate ciliation in multi-ciliated cells (Figure 4a). In a conserved transcriptional cascade, the inhibition of Notch signaling induces MCIDAS followed by the establishment of a ternary complex with E2F-4/5 and Dp1, which further activates downstream ciliary transcription factors, including RFX2 and FOXJ1, and controls the expression of core multi-ciliogenesis genes [18,19,71,72].

Furthermore, cluster IV revealed the interaction among the proteins involved in cAMP regulation, i.e., adenylate cyclase (ADCY3 or AC3) and phosphodiesterases (PDE5A and PDE4B) in cilia (Figure 4a). Ciliary beating is one of the crucial components for the accomplishment of an efficient mucociliary clearance whose malfunction causes several chronic airway diseases such as chronic bronchitis and cystic fibrosis. The intraciliary cAMP along with intracellular pH, Ca^2+^, and HCO_3_^−^ concentration regulates ciliary beat frequency (CBF) [272,273]. The cAMP-dependent activation of protein kinase A (PKA) causes the phosphorylation of an outer arm dynein light chain, which speeds up micro tubular sliding and CBF [274]. However, the involvement of adenylate cyclase in this process is not fully understood. The HCO_3_^−^ sensitive adenylate cyclase and cAMP also regulate the CFTR, which is crucial for innate defenses in the lung during exacerbations of airway diseases [275]. During *P. aeruginosa* and *K. pneumoniae* infections of the airways, the expression of IL-17A has been shown to become elevated, which in turn increases the secretion of HCO_3_^−^ in the airway lumen [276,277,278]. Jiang and co-workers also demonstrated the mechanism behind the cAMP-based regulation of ciliary hedgehog (Hh) signaling, where they reported that Gα(s)-coupled GPCR regulated ciliary cAMP negatively regulated Hh transcriptional activity in cilium [279].

Mucus cell hyperplasia/metaplasia and the increased size/frequency of submucosal gland causes an excessive production of mucus in asthma and COPD patients [280,281,282,283]. *MUC5AC* and MUC5B are the most abundantly found mucins in airway mucus secreted during inflammatory lung diseases [284,285,286]. Inflammatory cytokines secreted into the airways affected by lung diseases [287] are one of several stimuli that can promote mucin genes’ expression. Inflammatory cytokines that can affect mucin expression are categorized into pro-inflammatory (TNF-α and IL-1β), Th1 (IFN-γ), TH17 (IL-17A), and Th2 (IL-4, IL-9, and IL-13) cytokines [30]. TNF-α and IL-1β promote MUC5AC expression via a variety of mechanisms [288,289,290,291,292]. In one mechanism, TNF-α activates two different MAP kinases, i.e., ERK and p38, through distinct pathways that promote the downstream activation of cyclic AMP-responsive element binding protein (CREB). The CREB protein further binds to a CREB-responsive cis element present on the *MUC5AC* promoter and activates the expression of MUC5AC [289]. However, apart from the CREB-mediated activation of MUC5AC, in NCI-H292 cells, TNF-α can also promote MUC5AC expression through NF-kB [293]. The *MUC5AC* promoter also exhibits putative binding sites for NF-kB, which is considered a major transcriptional regulator of *MUC5AC* gene expression [294]. IL-4, IL-9, and IL-13 are also thought to affect the *MUC5AC* and *MUC5B* gene expression in airway epithelial cells, though their roles are controversial [287,295,296]. IL-4 and IL-13 bind to a common alpha receptor (IL-4Rα), activate Janus kinase (JAK) pathways followed by nuclear translocation of STAT6, and the further activation of STAT6-responsive genes. Chen et al. (2003) demonstrated that IL-6 and IL-17 can also upregulate both *MUC5B* and *MUC5AC*. In the primary airway epithelial cells, IL-17A indirectly upregulates *MUC5B* expression, which is partially dependent on the IL-6 and JAK2-dependent autocrine/paracrine loop. Furthermore, IL-6 controls the expression of *MUC5B* via the ERK MAP kinase pathway [295].

Cluster VI has an ACE2 protein that has been considered as an entry factor for SARS-CoV-2 [216,218]. It has been documented that SARS-CoV infection causes a reduction in cell membrane ACE2 concentration, and subsequently increases serum angiotensin II (Ang II), which results in severe lung inflammation followed by the development of acute respiratory distress syndrome (ARDS) [297]. Ang II produced by the action of ACE2 is a vasoconstrictor but can also act as a pro-inflammatory cytokine via angiotensin receptor type 1 (AT1R) [298]. The Ang II binding to AT1R activates NF-kB [298]. However, during inflammation, STAT3 is essential for the complete activation of the NF-kB pathway [299]. STAT3 is stimulated by the IL-6 family of cytokines [299]. ARDS-associated SARS-CoV infection is the result of the hyper-activation of the NF-kB pathway, which causes the release of pro-inflammatory cytokines such as IL-6, TNF-α, and chemokines produced by immune cells and non-immune cells. The SARS-CoV-dependent activation of NF-kB followed the MyD88 pathway through pattern recognition receptors (PRRs) [300].

Cluster VII showed the presence of another protein, i.e., cyclophilin A (PPIA), which has a critical role in SARS-CoV-2 infection (Figure 4a). PPIA promotes the interaction between non-structural protein 1 (nsp1) of SARS-CoV and CD147, which causes a reduction in the interferon responses in infected cells [301]. In lungs, a higher expression of CD147 was observed in both the epithelial tissues, as well as in innate and adaptive immune cells (macrophages, monocytes, ILCs, NK cells, T cells, and B cells), suggesting them as potential targets of infection via CD147 [302]. Cyclophilin/PPIA is also known to inhibit NF-ATs (nuclear factor of activated T-cells) activation in T cells, which ultimately causes the suppression of immune responses in infected cells [301,303]. Therefore, it was suggested that cyclophilin–CD147 complex formation is an important event for the local and systemic spread of SARS-CoV-2 via CD147 in a comparable way to TMPRSS2 and SLC6A19 for ACE2. Cluster VII also possess a calcineurin subunit B type 1 (PPP3R1; regulatory subunit) as an interacting partner (Figure 4a). Calcineurin is a Ca^2+^ and calmodulin regulated serine/threonine-protein phosphatase that is well known for its role in signal transduction during T-cell activation [304]. The cyclophilin–cyclosporine A complex inhibits the calcineurin-based dephosphorylation of NF-AT, which blocks T-cell-dependent cytokine transcription (for example IL-2) and T-cell activation [305,306]. A C-type lectin, CD209, was also observed in cluster V (Figure 4a), which mediates coronavirus infection by interacting with the S glycoprotein’s RBD [229]. Cluster VIII possesses NRP1, an alternate entry receptor for SARS-CoV-2, which helps in potentiating the viral infectivity [226,227]. NRP1 receptor binds to the sequence ‘RRAR’ at the S1 subunit’s C-terminus, and its expression was found to be upregulated in patients with COVID-19 [228].

Furthermore, a partial interactome of SARS-CoV-2 and host proteins was extracted from the Network Data Exchange (NDEx) public server (www.ndexbio.org) [307,308,309] representing the IntAct/IMEx Coronavirus Dataset [310] to understand the point of interactions of SARS-CoV-2 proteins in the molecular machinery of mucociliary clearance (Figure 4b). The extracted viral–host protein interaction network was visualized in Cytoscape 3.7.2 (with CyNDEx-2 application) with the Compound Spring Embedder (CoSE) layout (the detailed methodology is present in the Supplementary Material, M&M S1). The viral–host interactome showed the interaction of SARS-CoV-2 components with seven proteins, i.e., ACE2, PPIA, CDK1, MCM3, RAB8A, NRP1, and CD209, belonging to predicted molecular machinery of mucociliary clearance (Figure 4b). The host ACE2 receptor protein behaves as the entry point for SARS-CoV-2. For invading host target cells, the SARS-CoV-2 spike (S) protein binds to an ACE2 receptor and is subsequently primed by a TMPRSS2. The TMPRSS2, a type II transmembrane serine protease, further cleaves the S protein, resulting in the fusion of viral and host lysosomal membranes [218]. The viral–host interactome also showed a connection between spike protein (S), ACE2 receptor and heparin (Figure 4b). Clausen and co-workers performed a docking analysis and demonstrated that the spike protein, ACE2 receptor, and heparin form a ternary complex where heparin broadens the RBD’s open conformation that associates with ACE2 [230]. Targeting the heparin/heparan sulfate-mediated enhancement of binding to ACE2 could be considered as a potential target for developing therapy for inhibiting viral adhesion. Though not revealed from the viral–host interactome, an alternate mechanism for SARS-CoV-2 invasion has also been deciphered recently in tissues with a low or absent ACE2 expression. The NRP1 host receptor has been reported to bind to a polybasic sequence ‘RRAR’ located at the S1–S2 junction in the S glycoprotein that helps in SARS-CoV-2 entry [224,226,227]. As discussed in the previous section, PPIA or cyclophilin is important for the local and systemic spread of SARS-CoV-2 via CD147. PPIA is known to promote the interaction between SARS-CoV nsp1 and CD147, which causes a reduction in the interferon responses in infected cells [301]. In the viral–host interactome, PPIA also interacts with NendoU (orf1ab polyprotein) and the SARS-CoV-2 nucleocapsid (N) protein (Figure 4b). Luo and co-workers also demonstrated the SARS-CoV nucleocapsid protein binding to human cyclophilin (hCypA or PPIA) with a high affinity [311]. A hypothetical protein with an unknown function, named y14_sars2 (ORF14 of SARS-CoV-2) in the viral–host network, was found to interact with the CDK1 and MCM3 (Figure 4b). CDK1 activity gets considerably diminished upon SARS-CoV-2 infection and causes an S/G2 phase arrest that supports viral replication by ensuring a sufficient resource of nucleotides, as well as other essential host DNA repair/replication proteins [312]. In addition, MCM3 also interacts with the non-structural proteins (nsp3, nsp7, nsp8, nsp10, and RdRP) (Figure 4b). The viral non-structural proteins are cleaved products of polyproteins ORF1a and ORF1ab, and they are involved in the formation of viral replication/transcription complex (RTC). Virus infection upregulates the TGFβ pathway, which modulates cell survival, motility, innate immune responses, and ultimately leads to fibrosis (hallmarks of COVID-19) [313,314]. The viral–host network also showed the interaction between ORF3a (ap3a_sars) and TGFBR2 (one of the TGFβ-associated factors) (Figure 4b). Stukalov and co-workers performed a network diffusion analysis and showed the association between ORF3, TGFβ-associated factors (namely TGFB1, TGFB2, LTBP1, TGFBR2, FURIN, and BAMBI), and the virus-mediated upregulation of fibrinogens, fibronectin, SERPINE1, and integrin(s) [315]. As discussed earlier, CD209 can mediate coronavirus infection by interacting with the RBD of the S glycoprotein [229]. The viral–host interactome also showed the CD209 interaction with the spike protein (Figure 4b).

The overall network has provided the probable pathway involved in the regulation of mucociliary clearance and associated processes following SARS-CoV-2 infection. Host proteins like ACE2, PPIA, NRP1, and CD209 act as the entry points and infection targets upon SARS-CoV-2 invasion. The invading SARS-CoV-2 may further activate the host immune system, which in turn activates mucins (MUC5AC and MUC5B) and causes exessive mucus production, a condition commonly found in inflammatory repiratory diseases. SARS-CoV-2 infection might influence the cAMP-dependent ciliary beat frequency critical for an efficient mucociliary clearance. SARS-CoV-2 might also impact the host proteins controlling the cell cycle (CDK1 and MCM3), IFT machinery (IFT88, IFT20, and BBS1) and ciliogenesis (DYNLL1, DYNC2LL1, DYNC2H1, FOXJ1, RFX3, and MCIDAS). Thus, based on the protein–protein network analysis (Figure 4a,b), we have predicted the probable regulatory mechanism and the points of interactions for viral proteins in the molecular machinery of host mucociliary clearance upon SARS-CoV-2 infection.

### 4.3. Modulation of Gene Expression of the MCC System upon SARS-CoV-2 Infection

**Figure 5:**
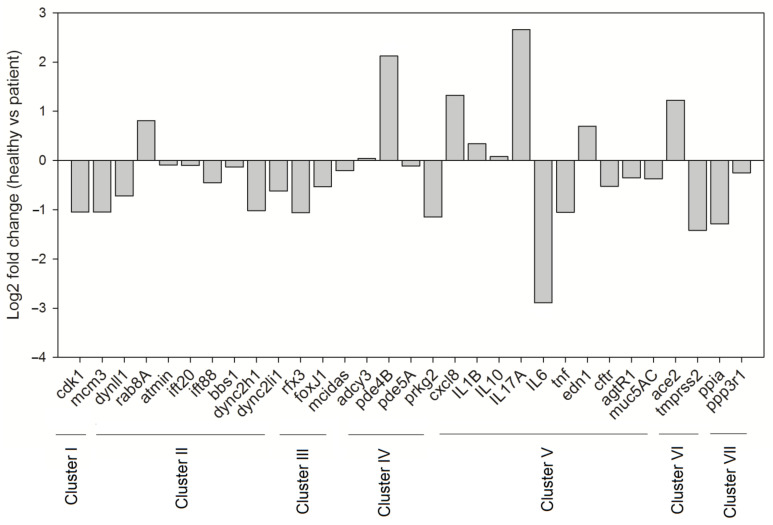
Differential expression (log2 fold change) of mucociliary clearance related gene transcripts in SARS-CoV-2-infected patients relative to healthy individuals. Clusters I–V represent genes associated with cell cycle regulation, IFT machinery, cilia biogenesis and regulation, cAMP regulation, and immune defense, respectively, whereas cluster VI and VII represent genes controlling SARS-CoV-2 entry and infection, respectively.

The shotgun RNA sequencing profiles of nasopharyngeal swabs from SARS-CoV-2-infected patients versus healthy individuals revealed the in vivo transcriptional response to infection by the coronavirus. The R package DESeq 2 (Version 1.26.0) [316] was utilized to calculate the normalized gene read counts and the differential expression (log2 fold change) of mucociliary clearance related genes in SARS-CoV-2-infected and healthy individuals (detailed methodology is present in the Supplementary Material, M&M S2) using NCBI GEO RNA-seq dataset published by Lieberman and co-workers (GEO accession: GSE152075) [317]. Figure 5 depicts the differential expression of gene transcripts related to the mucociliary clearance machinery. Most of the selected gene transcripts showed differential expression (log2 fold change > 1), but the changes were not found to be significant (*padj* > 0.05) in any case (Table S3). The expression data reported by Lieberman et al. (2020) also revealed an unexpected downregulation of the *MUC5AC* gene and a few inflammatory cytokines (IL-6 and TNF) in SARS-CoV-2-infected patients (Figure 5). These unexpected observations can be explained by the fact that nasopharyngeal swabs from patient and healthy individuals were collected for RNA-seq analysis, which is not an ideal or sensitive anatomic location to test for systemic inflammation. Therefore, systematic and detailed studies are required to understand the complete mechanism of the transcriptional regulation of mucociliary clearance related genes in SARS-CoV-2-infected individuals, which is a rather unexplored area till date.

## 5. Molecular Basis of Repurposing of Drugs for Mitigating SARS-CoV-2-Induced Lung Cilia and MCC Dysfunctions

At this critical time of an ongoing pandemic, researchers worldwide have delved into the development of an effective therapeutic agent to tackle COVID-19. Until an effective antidote is developed, the repurposing of existing drugs seems to be the most viable strategy to manage COVID-19. As SARS-CoV-2 affects the respiratory system and destroys the ciliated cells, an investment in existing drugs that improve pulmonary function by enhancing the mucociliary clearance system seems necessary. Bronchitol^®^ (Pharmaxis Ltd.) is an inhaled dried powder preparation of mannitol that is used as a management strategy for adult patients diagnosed with cystic fibrosis [318]. It targets the lungs and acts by increasing the osmolarity of the periciliary fluid that helps to hydrate the lung surface [319]. This augments mucociliary clearance and also improves cough clearance, thereby sloughing off the mucus obstruction and preventing the associated pulmonary exacerbations seen in cystic fibrosis. β-adrenergic agonists increase the ciliary beat frequency and improve the mucociliary clearance rate in respiratory diseases like asthma and cystic fibrosis [320]. Davis and co-workers reported that terbutaline increased the active Cl^−^ transport across the canine tracheal airway epithelium [321]. This effects the passive movement of water that hydrates the epithelial surface and may result in MCC augmentation. The recombinant form of human DNase I (Pulmozyme^®^, dornase alfa) hydrolyses the DNA in the purulent secretions from the airways of CF patients, reduces mucus viscoelasticity, and improves pulmonary function [322,323]. The inhalation of amiloride (Na^+^ channel blocker) has been found to improve sputum viscosity and mucus clearance, as well as to increase cough-mediated sputum expectoration in CF patients, thus contributing to a delay in lung function [324,325]. Denufosol, a P2Y(2) receptor agonist, functions by bypassing the CFTR ion channels and activating alternate Cl^−^ ion channels. This augments epithelial surface hydration, which is directly linked to an effective mucus clearance mediated by efficient ciliary beating [326].

## 6. Concluding Remarks

The human respiratory system sustains life via the regular uptake of oxygen from the environment. However, in the process, pollutants and noxious pathogens often invade the lungs along with the vital oxygen. To evade and overcome such respiratory assaults, many embedded guards like the cilia and the mucus lining exist in the MCC machinery. Currently, we have been confronted by a challenge posed by the novel virus, SARS-CoV-2. COVID-19 is an acute infectious respiratory disease that spreads via the nasal or oral route and colonizes the respiratory system. As medical workers and scientists probe further details of SARS-CoV-2 and the ensuing diseased state, people of elderly age and with pre-existing health conditions have been recognized to be more vulnerable to infection and to develop a severe form of the illness. The epithelial cilia in conjunction with respiratory mucus are the primary barriers against such diseases that target the human airways. Consequently, genetic or acquired aberrations in these defense arrangements like asthma, cystic fibrosis, COPD, and ciliopathies like primary ciliary dyskinesia are perilous because they provide a suitable environment for susceptibility to and fatality from COVID-19. Thus, such impairments in the mucociliary escalator should be stringently identified and managed, and surveillance should be intensified in the current scenario until an effective antiviral intervention is developed.

## Data Availability

Most of the data presented in this study are available in this article and/or supplementary material. Restrictions may apply to the availability of some data as the same was obtained from third party and are available from the authors/with the permission of third party as referred in this manuscript.

## Supplementary Materials

The following are available online at https://www.mdpi.com/2079-7737/10/2/95/s1, Table S1: Table showing string output in terms of various evidence types (GO, local network cluster, KEGG pathway, reactome pathways, annotated keywords and protein domains) contributing to the molecular network of the lung cilia and MCC machinery regulating mucociliary clearance in humans. The string output depicts the network statistics as well as the false discovery rate (FDR) values for each evidence. The evidences with FDR value < 0.05 were considered significant. Table S2: String output in terms of various evidence types (GO, local network cluster, KEGG pathway, reactome pathways, annotated keywords and protein domains) contributing to the network representing the SARS-CoV-2 infection induced implications in the molecular machinery regulating mucociliary clearance. The string output depicts the network statistics as well as the false discovery rate (FDR) values for each evidence. The evidences with FDR value < 0.05 were considered significant. Table S3: List of differentially expressed transcripts related to mucociliary clearance machinery in SARS-CoV-2 infected patients relative to healthy individuals.
