# Supplementary material for: Mucociliary Respiratory Epithelium Integrity in Molecular Defense and Susceptibility to Pulmonary Viral Infections
Source: Biology (Basel). 2021 Jan 29;10(2):95. doi: 10.3390/biology10020095 (PMC7911113; doi:10.3390/biology10020095)
Supplement: Supplementary file 1 [file biology-10-00095-s001.pdf]

Supplementary Material

Mucociliary Respiratory Epithelium Integrity in Molecular Defense and Susceptibility to Pulmonary Viral Infections

Adivitiya [1], Manish Singh Kaushik [1], Soura Chakraborty [1], Shobi Veleri [2] and Suneel Kateriya [1,*]

[1] Laboratory of Optobiology, School of Biotechnology, Jawaharlal Nehru University, New Delhi-110067, India
[2] Drug Safety Division, ICMR-National Institute of Nutrition, Hyderabad-500007, India
* Correspondence: skateriya@jnu.ac.in

M&M S1. Protein-protein network analysis

To study the molecular mechanism regulating the mucociliary clearance and changes occurring in this machinery following SARS-CoV-2 infection, the proteins reported to be involved either directly or indirectly in this process were selected by referring to relevant research papers and subjected to *in silico* protein-protein interaction analysis. We have included both experimentally validated and predicted protein-protein interactions in our analysis of the protein network. The network of the selected proteins was constructed using String version 11 (Academic Consortium 2020) [1]. The online STRING database integrates available resources having information related to protein–protein interactions [direct (physical) as well as indirect (functional) interactions] for constructing a comprehensive network [1]. STRING, as a tool, utilizes the classification systems based on Gene Ontology and KEGG, high-throughput text-mining as well as on hierarchical clustering of the association network. STRING brings in the functional association between two proteins responsible for the onset of a specific biological function. The evidences supporting the association between two proteins are retrieved from different and independent channels (data sources) available for genomic context information, co-expression, text-mining, biochemical/genetic data, and previously curated pathway and protein-complex knowledge [1]. Each data source generates separate interaction scores as well as the FDR (false discovery rate) value, which represent confidence (on a scale of zero to one) and significance of the association, respectively. The FDR (false discovery rate) approach was employed to assess the significance of the series of tests performed, where *p-value* represents the probability of false positives on a single test [2]. The STRING output was further analyzed with Cytoscape 3.7.2 (San Diego, CA, USA) by employing *Betweenness* centrality algorithm [3]. The *Betweenness* Centrality algorithm determines the shortest path between each pair of nodes in a network [4]. The nodes with high betweenness value actually have the determining hold over the network. Furthermore, clustering of the protein network was performed by employing Markov clustering (MCL) algorithm [5]. Finally, a partial interactome of SARS-CoV-2 and host proteins was extracted from the Network Data Exchange (NDEx) public server (www.ndexbio.org) [6-8] representing the IntAct/IMEx Coronavirus Dataset [9] to understand the point of interactions of SARS-CoV-2 proteins in the molecular machinery of mucociliary clearance. The extracted viral-host protein interaction network has been visualized in Cytoscape 3.7.2 (with CyNDEx-2 application) with Compound Spring Embedder (CoSE) layout [3].

M&M S2. In-silico expression analysis

We have analyzed the expression profile of mucociliary clearance genes from SARS-CoV-2 infected patients versus healthy individuals. We have used RNA-seq dataset (GEO accession: GSE152075) generated through high throughput sequencing of nasopharyngeal swab samples from SARS-CoV-2 infected and healthy individuals [10]. The DESeq 2 (Version 1.26.0) [11] normalization was further performed in R studio (version 4.0.3, http://www.rstudio.com/) [12] to calculate the differential expression of transcripts related to mucociliary clearance. Batch correction was performed, log2 fold changes of each genes and

corresponding *p-values* were calculated (Table S3). Log2 fold change was plotted to analyze the impact of SARS-CoV-2 infection on the expression of genes related to host mucociliary clearance system.

Table S1: Table showing string output in terms of various evidence types (GO, local network cluster, KEGG pathway, reactome pathways, annotated keywords and protein domains) contributing to the molecular network of the lung cilia and MCC machinery regulating mucociliary clearance in humans. The string output depicts the network statistics as well as the false discovery rate (FDR) values for each evidence. The evidences with FDR value < 0.05 were considered significant.

**Network statistics**		
Number of nodes	39	
Number of edges	153	
Average node degree	7.65	
Avg. local clustering coefficient	0.69	
Expected number of edges	22	
PPI enrichment *p-value*	< 1.0e-16	
**Term ID**	**Term description**	**False discovery rate (FDR)**
Biological Process (GO)		
GO:0060271	cilium assembly	1.84E-18
GO:0070925	organelle assembly	1.19E-16
GO:0006270	DNA replication initiation	1.46E-14
GO:0044772	mitotic cell cycle phase transition	1.98E-14
GO:0030030	cell projection organization	2.75E-14
GO:0006261	DNA-dependent DNA replication	1.29E-13
GO:0000082	G1/S transition of mitotic cell cycle	2.57E-13
GO:0000278	mitotic cell cycle	2.57E-13
GO:0007049	cell cycle	4.80E-13
GO:0006260	DNA replication	8.81E-13
GO:1903047	mitotic cell cycle process	8.81E-13
GO:0022402	cell cycle process	3.00E-12
GO:0006996	organelle organization	3.43E-12
GO:0022607	cellular component assembly	4.92E-12
GO:1903251	multi-ciliated epithelial cell differentiation	2.28E-09
GO:0016043	cellular component organization	1.30E-08
GO:0006259	DNA metabolic process	1.33E-08
GO:0098534	centriole assembly	9.67E-08
GO:0007017	microtubule-based process	1.13E-07
GO:0044458	motile cilium assembly	1.34E-07
GO:0034645	cellular macromolecule biosynthetic process	2.09E-07
GO:0006268	DNA unwinding involved in DNA replication	3.39E-07
GO:0031023	microtubule organizing center organization	1.57E-06
GO:0000226	microtubule cytoskeleton organization	4.00E-06
GO:0090304	nucleic acid metabolic process	1.02E-05

GO:0044260	cellular macromolecule metabolic process	1.35E-05
GO:0009888	tissue development	1.40E-05
GO:0006139	nucleobase-containing compound metabolic process	2.85E-05
GO:0060429	epithelium development	3.18E-05
GO:0006275	regulation of DNA replication	6.15E-05
GO:0007507	heart development	0.00018
GO:1901990	regulation of mitotic cell cycle phase transition	0.00021
GO:0009987	cellular process	0.00028
GO:1901992	positive regulation of mitotic cell cycle phase transition	0.00032
GO:0060972	left/right pattern formation	0.00041
GO:0007010	cytoskeleton organization	0.00044
GO:0140056	organelle localization by membrane tethering	0.00051
GO:0051276	chromosome organization	0.00064
GO:0007346	regulation of mitotic cell cycle	0.00068
GO:0006807	nitrogen compound metabolic process	0.00081
GO:0000083	regulation of transcription involved in G1/S transition of mitotic cell cycle	0.00086
GO:0071163	DNA replication preinitiation complex assembly	0.00091
GO:0030855	epithelial cell differentiation	0.001
GO:0097711	ciliary basal body-plasma membrane docking	0.0011
GO:0021943	formation of radial glial scaffolds	0.0013
GO:0051726	regulation of cell cycle	0.0015
GO:0098535	de novo centriole assembly involved in multi-ciliated epithelial cell differentiation	0.0018
GO:0007368	determination of left/right symmetry	0.0019
GO:0031503	protein-containing complex localization	0.0019
GO:0035735	intraciliary transport involved in cilium assembly	0.0019
GO:0048856	anatomical structure development	0.0019
GO:0001714	endodermal cell fate specification	0.0021
GO:0009653	anatomical structure morphogenesis	0.0021
GO:0044237	cellular metabolic process	0.0021
GO:0044238	primary metabolic process	0.0022
GO:0051640	organelle localization	0.0028
GO:0071103	DNA conformation change	0.0032
GO:1902017	regulation of cilium assembly	0.0032
GO:0030154	cell differentiation	0.0038
GO:0031570	DNA integrity checkpoint	0.0039
GO:0090068	positive regulation of cell cycle process	0.0039
GO:0010389	regulation of G2/M transition of mitotic cell cycle	0.004
GO:0071704	organic substance metabolic process	0.004

GO:0098969	neurotransmitter receptor transport to postsynaptic membrane	0.004
GO:0001947	heart looping	0.0041
GO:0072659	protein localization to plasma membrane	0.0045
GO:0035563	positive regulation of chromatin binding	0.0051
GO:0001708	cell fate specification	0.0064
GO:0007224	smoothened signaling pathway	0.0064
GO:0051302	regulation of cell division	0.0067
GO:0000076	DNA replication checkpoint	0.0075
GO:0007098	centrosome cycle	0.0075
GO:0007099	centriole replication	0.0075
GO:0045786	negative regulation of cell cycle	0.0076
GO:0055007	cardiac muscle cell differentiation	0.0076
GO:0099632	protein transport within plasma membrane	0.0081
GO:0032465	regulation of cytokinesis	0.0086
GO:0048598	embryonic morphogenesis	0.0094
GO:0051668	localization within membrane	0.0116
GO:0051052	regulation of DNA metabolic process	0.0121
GO:0010971	positive regulation of G2/M transition of mitotic cell cycle	0.013
GO:0048169	regulation of long-term neuronal synaptic plasticity	0.0139
GO:0048513	animal organ development	0.0148
GO:0051984	positive regulation of chromosome segregation	0.0148
GO:0006928	movement of cell or subcellular component	0.015
GO:0007389	pattern specification process	0.0154
GO:0002009	morphogenesis of an epithelium	0.016
GO:0000086	G2/M transition of mitotic cell cycle	0.0216
GO:0008283	cell population proliferation	0.024
GO:0007018	microtubule-based movement	0.0241
GO:0008156	negative regulation of DNA replication	0.0241
GO:0045740	positive regulation of DNA replication	0.0247
GO:0048468	cell development	0.0259
GO:0140014	mitotic nuclear division	0.0265
GO:2000785	regulation of autophagosome assembly	0.0266
GO:0051301	cell division	0.0277
GO:1905515	non-motile cilium assembly	0.029
GO:0090307	mitotic spindle assembly	0.0302
GO:2000045	regulation of G1/S transition of mitotic cell cycle	0.0319
GO:0007080	mitotic metaphase plate congression	0.0321
GO:0003002	regionalization	0.0337
GO:0007399	nervous system development	0.0337
GO:0090329	regulation of DNA-dependent DNA replication	0.0342

GO:0006974	cellular response to DNA damage stimulus	0.0343
GO:0090102	cochlea development	0.035
GO:0006355	regulation of transcription, DNA-templated	0.0352
GO:0043433	negative regulation of DNA-binding transcription factor activity	0.0352
GO:2000112	regulation of cellular macromolecule biosynthetic process	0.0352
GO:0007369	gastrulation	0.0353
GO:0050794	regulation of cellular process	0.0368
GO:0098900	regulation of action potential	0.0375
GO:0030071	regulation of mitotic metaphase/anaphase transition	0.0382
GO:0002758	innate immune response-activating signal transduction	0.0397
GO:0051716	cellular response to stimulus	0.0401
GO:0019219	regulation of nucleobase-containing compound metabolic process	0.0403
GO:0048731	system development	0.0405
GO:0035019	somatic stem cell population maintenance	0.0417
GO:0048839	inner ear development	0.043
GO:0002064	epithelial cell development	0.0441
GO:0016266	O-glycan processing	0.0467
GO:0045944	positive regulation of transcription by RNA polymerase II	0.0477
GO:0006977	DNA damage response, signal transduction by p53 class mediator resulting in cell cycle arrest	0.0488
Molecular Function (GO)		
GO:0003688	DNA replication origin binding	0.00076
GO:0004386	helicase activity	0.001
GO:1990837	sequence-specific double-stranded DNA binding	0.001
GO:0003677	DNA binding	0.0016
GO:0003697	single-stranded DNA binding	0.0016
GO:0003678	DNA helicase activity	0.002
GO:0031489	myosin V binding	0.0109
GO:0017111	nucleoside-triphosphatase activity	0.0137
GO:1901363	heterocyclic compound binding	0.0169
GO:0097159	organic cyclic compound binding	0.0191
GO:0035639	purine ribonucleoside triphosphate binding	0.0234
GO:0003682	chromatin binding	0.0267
GO:0032555	purine ribonucleotide binding	0.0267
GO:0097367	carbohydrate derivative binding	0.0267
GO:0000976	transcription regulatory region sequence-specific DNA binding	0.0394

GO:0000977	RNA polymerase II transcription regulatory region sequence-specific DNA binding	0.0483
GO:0001228	DNA-binding transcription activator activity, RNA polymerase II-specific	0.0483
<u>Cellular component (GO)</u>		
GO:0005814	centriole	1.17E-09
GO:0042555	MCM complex	3.59E-09
GO:0005815	microtubule organizing center	4.36E-08
GO:0015630	microtubule cytoskeleton	1.30E-07
GO:0043232	intracellular non-membrane-bounded organelle	3.94E-07
GO:0005856	cytoskeleton	9.50E-07
GO:0043229	intracellular organelle	4.06E-06
GO:0005929	cilium	4.01E-05
GO:0005622	intracellular	6.57E-05
GO:0036064	ciliary basal body	6.57E-05
GO:0030992	intraciliary transport particle B	0.00019
GO:0005654	nucleoplasm	0.0002
GO:0043231	intracellular membrane-bounded organelle	0.0003
GO:0005634	nucleus	0.00043
GO:0031981	nuclear lumen	0.00043
GO:0097546	ciliary base	0.00043
GO:0005813	centrosome	0.00045
GO:0043227	membrane-bounded organelle	0.00045
GO:0098536	deuterosome	0.0008
GO:0005694	chromosome	0.00096
GO:0070013	intracellular organelle lumen	0.001
GO:0097542	ciliary tip	0.001
GO:0005664	nuclear origin of replication recognition complex	0.0016
GO:0042995	cell projection	0.0028
GO:0044292	dendrite terminus	0.0032
GO:0000228	nuclear chromosome	0.0039
GO:0005623	cell	0.004
GO:0120025	plasma membrane bounded cell projection	0.0072
GO:0032991	protein-containing complex	0.0079
GO:0005930	axoneme	0.0084
GO:0043596	nuclear replication fork	0.0093
GO:0032391	photoreceptor connecting cilium	0.0109
GO:0097730	non-motile cilium	0.0124
GO:0031514	motile cilium	0.0269
GO:0005802	trans-Golgi network	0.0401
GO:0005794	Golgi apparatus	0.041
GO:0099568	cytoplasmic region	0.041

GO:0055038	recycling endosome membrane	0.0453
Local network Cluster (String)		
CL:12805	Activation of the pre-replicative complex	2.39E-18
CL:20274	mixed, incl. Ciliopathy, and dynein complex	8.94E-10
CL:12807	nuclear origin of replication recognition complex, and MCM N-terminal domain	4.33E-09
CL:20560	RFX DNA-binding domain, and multi-ciliated epithelial cell differentiation	6.94E-09
CL:12835	Magnesium chelatase, subunit ChlI	9.89E-06
CL:20430	intraciliary transport particle B	0.0001
CL:20562	RFX1 transcription activation region, and Poxvirus D5 protein-like	0.00098
CL:26973	Defective GALNT3 causes familial hyper phosphatemic tumoral calcinosis (HFTC)	0.00098
CL:6326	POU5F1 (OCT4), SOX2, NANOG repress genes related to differentiation, and RNA polymerase II sequence-specific DNA-binding transcription factor recruiting activity	0.00098
CL:20275	Ciliopathy, and dynein complex	0.0019
CL:20433	intraciliary transport particle B	0.0021
CL:19898	ciliary basal body-plasma membrane docking, and gamma-tubulin complex	0.0055
CL:19904	ciliary basal body-plasma membrane docking	0.0164
KEGG Pathways		
hsa04110	Cell cycle	2.27E-10
hsa03030	DNA replication	4.45E-07
Reactome Pathways		
HSA-68962	Activation of the pre-replicative complex	1.35E-16
HSA-69206	G1/S Transition	5.15E-15
HSA-176187	Activation of ATR in response to replication stress	8.66E-13
HSA-68867	Assembly of the pre-replicative complex	8.66E-13
HSA-69278	Cell Cycle, Mitotic	8.66E-13
HSA-68949	Orc1 removal from chromatin	4.88E-11
HSA-69481	G2/M Checkpoints	3.98E-10
HSA-69242	S Phase	5.75E-10
HSA-176974	Unwinding of DNA	1.67E-09
HSA-5617833	Cilium Assembly	3.11E-09
HSA-539107	Activation of E2F1 target genes at G1/S	5.15E-06
HSA-69205	G1/S-Specific Transcription	5.15E-06
HSA-5620912	Anchoring of the basal body to the plasma membrane	1.71E-05

HSA-68827	CDT1 association with the CDC6:ORC:origin complex	5.83E-05
HSA-1538133	G0 and Early G1	0.00026
HSA-2565942	Regulation of PLK1 Activity at G2/M Transition	0.00026
HSA-2892245	POU5F1 (OCT4), SOX2, NANOG repress genes related to differentiation	0.00031
HSA-5620920	Cargo trafficking to the periciliary membrane	0.0013
HSA-5620924	Intraflagellar transport	0.0013
HSA-113507	E2F-enabled inhibition of pre-replication complex formation	0.0015
HSA-68689	CDC6 association with the ORC:origin complex	0.0021
HSA-2892247	POU5F1 (OCT4), SOX2, NANOG activate genes related to proliferation	0.0024
HSA-380259	Loss of Nlp from mitotic centrosomes	0.0026
HSA-8854518	AURKA Activation by TPX2	0.0027
HSA-1362300	Transcription of E2F targets under negative control by p107 (RBL1) and p130 (RBL2) in complex with HDAC1	0.0035
HSA-380270	Recruitment of mitotic centrosome proteins and complexes	0.0035
HSA-5083625	Defective GALNT3 causes familial hyper phosphatemic tumoral calcinosis (HFTC)	0.0035
HSA-5083632	Defective C1GALT1C1 causes Tn polyagglutination syndrome (TNPS)	0.0035
HSA-5083636	Defective GALNT12 causes colorectal cancer 1 (CRCS1)	0.0035
HSA-6804114	TP53 Regulates Transcription of Genes Involved in G2 Cell Cycle Arrest	0.0035
HSA-1362277	Transcription of E2F targets under negative control by DREAM complex	0.0038
HSA-380320	Recruitment of NuMA to mitotic centrosomes	0.0041
HSA-5620916	VxPx cargo-targeting to cilium	0.0044
HSA-977068	Termination of O-glycan biosynthesis	0.0047
HSA-5621480	Dectin-2 family	0.0061
HSA-3769402	Deactivation of the beta-catenin transactivating complex	0.0137
HSA-8854214	TBC/RABGAPs	0.0147
HSA-8873719	RAB geranyl geranylation	0.0279
HSA-201681	TCF dependent signaling in response to WNT	0.028
HSA-1445148	Translocation of SLC2A4 (GLUT4) to the plasma membrane	0.0314
Annotated Keywords (UniProt)		
KW-0970	Cilium biogenesis/degradation	8.68E-19
KW-0235	DNA replication	4.96E-13

KW-0131	Cell cycle	3.35E-11
KW-1186	Ciliopathy	1.24E-05
KW-0969	Cilium	1.79E-05
KW-0539	Nucleus	3.08E-05
KW-0206	Cytoskeleton	4.10E-05
KW-0347	Helicase	0.00013
KW-0238	DNA-binding	0.00018
KW-0175	Coiled coil	0.0005
KW-0990	Primary ciliary dyskinesia	0.00055
KW-0966	Cell projection	0.00059
KW-0242	Dwarfism	0.0021
KW-0010	Activator	0.0025
KW-0832	Ubl conjugation	0.0037
KW-0597	Phosphoprotein	0.004
KW-0963	Cytoplasm	0.004
KW-0132	Cell division	0.005
KW-1017	Isopeptide bond	0.0091
KW-0498	Mitosis	0.0098
KW-0547	Nucleotide-binding	0.0098
KW-9995	Disease	0.0098
KW-0225	Disease mutation	0.0184
Protein domains (Pfam)		
PF01078	Magnesium chelatase, subunit ChlI	7.24E-10
PF14551	MCM N-terminal domain	7.24E-10
PF17207	MCM OB domain	7.24E-10
PF00493	MCM2/3/5 family	7.81E-10
PF07726	ATPase family associated with various cellular activities (AAA)	3.18E-06
PF07412	Geminin	0.00023
PF07728	AAA domain (dynein-related subfamily)	0.00048
PF13330	Mucin-2 protein WxxW repeating region	0.00061
PF01826	Trypsin Inhibitor like cysteine rich domain	0.0017
PF08742	C8 domain	0.0017
PF00094	von Willebrand factor type D domain	0.0025
PF08477	Ras of Complex, Roc, domain of DAP kinase	0.0211
PF00250	Forkhead domain	0.0217
PF00025	ADP-ribosylation factor family	0.0221
PF00071	Ras family	0.0221
Protein domains (SMART)		
SM00350	minichromosome maintenance proteins	9.36E-10
SM00382	ATPases associated with a variety of cellular activities	0.0031

SM00832	This domain contains 8 conserved cysteine residues, but this family only contains 7 of them to overlaps with other domains. It is found in disease-related proteins including von Willebrand factor, Alpha tectorin, Zonadhesin and Mucin.	0.0031
SM00216	von Willebrand factor (vWF) type D domain	0.0037
SM00041	C-terminal cystine knot-like domain (CTCK)	0.0049
SM00214	von Willebrand factor (vWF) type C domain	0.0116
SM00339	FORKHEAD	0.0187
SM00175	Rab subfamily of small GTPases	0.0255

GO: gene ontology; KEGG: Kyoto encyclopedia of gene and genomes; pfam: Protein family database; SMART: Simple modular architecture research tool.

Table S2: String output in terms of various evidence types (GO, local network cluster, KEGG pathway, reactome pathways, annotated keywords and protein domains) contributing to the network representing the SARS-CoV-2 infection induced implications in the molecular machinery regulating mucociliary clearance. The string output depicts the network statistics as well as the false discovery rate (FDR) values for each evidence. The evidences with FDR value < 0.05 were considered significant.

**Network Statistics**		
Number of nodes	36	
Number of edges	109	
Average node degree	6.06	
Avg. local clustering coefficient	0.583	
Expected number of edges	38	
PPI enrichment p-value	< 1.0e-16	
**Term ID**	**Term description**	**False discovery rate (FDR)**
Biological Process (GO)		
GO:0006928	movement of cell or sub-cellular component	7.96E-10
GO:0060271	cilium assembly	7.96E-10
GO:0120031	plasma membrane bounded cell projection assembly	7.96E-10
GO:0051179	localization	1.25E-08
GO:0051240	positive regulation of multicellular organismal process	1.50E-08
GO:0032103	positive regulation of response to external stimulus	2.17E-08
GO:1903530	regulation of secretion by cell	3.57E-08
GO:0032879	regulation of localization	4.93E-08
GO:0051049	regulation of transport	5.79E-08
GO:0051094	positive regulation of developmental process	6.33E-08
GO:1901701	cellular response to oxygen-containing compound	6.33E-08
GO:0048870	cell motility	6.95E-08
GO:0050714	positive regulation of protein secretion	8.64E-08

GO:0040011	locomotion	9.29E-08
GO:0032101	regulation of response to external stimulus	9.65E-08
GO:0051223	regulation of protein transport	1.03E-07
GO:0048518	positive regulation of biological process	1.04E-07
GO:0051222	positive regulation of protein transport	1.19E-07
GO:1903532	positive regulation of secretion by cell	1.19E-07
GO:0120036	plasma membrane bounded cell projection organization	1.81E-07
GO:0010243	response to organonitrogen compound	2.44E-07
GO:0002684	positive regulation of immune system process	2.56E-07
GO:0050900	leukocyte migration	2.57E-07
GO:0048584	positive regulation of response to stimulus	2.84E-07
GO:0050708	regulation of protein secretion	2.91E-07
GO:0035690	cellular response to drug	3.49E-07
GO:0042127	regulation of cell population proliferation	4.43E-07
GO:0002682	regulation of immune system process	5.92E-07
GO:0071222	cellular response to lipopolysaccharide	7.31E-07
GO:0048583	regulation of response to stimulus	7.35E-07
GO:0042129	regulation of T cell proliferation	8.51E-07
GO:0016477	cell migration	8.68E-07
GO:0071396	cellular response to lipid	8.68E-07
GO:0035735	intraciliary transport involved in cilium assembly	1.07E-06
GO:0045766	positive regulation of angiogenesis	1.22E-06
GO:2000379	positive regulation of reactive oxygen species metabolic process	1.47E-06
GO:0048522	positive regulation of cellular process	1.54E-06
GO:2000377	regulation of reactive oxygen species metabolic process	1.54E-06
GO:0008284	positive regulation of cell population proliferation	1.73E-06
GO:0045765	regulation of angiogenesis	1.97E-06
GO:0051050	positive regulation of transport	1.97E-06
GO:1903037	regulation of leukocyte cell-cell adhesion	1.97E-06
GO:0060326	cell chemotaxis	2.19E-06
GO:0010469	regulation of signaling receptor activity	3.03E-06
GO:0032496	response to lipopolysaccharide	3.03E-06
GO:0032675	regulation of interleukin-6 production	3.10E-06
GO:0051704	multi-organism process	3.50E-06
GO:0022603	regulation of anatomical structure morphogenesis	3.54E-06
GO:0030335	positive regulation of cell migration	4.16E-06
GO:0001936	regulation of endothelial cell proliferation	4.30E-06
GO:0030155	regulation of cell adhesion	5.01E-06
GO:0002687	positive regulation of leukocyte migration	5.19E-06
GO:0002706	regulation of lymphocyte mediated immunity	6.56E-06

GO:0007507	heart development	6.74E-06
GO:0071417	cellular response to organonitrogen compound	6.74E-06
GO:0002822	regulation of adaptive immune response based on somatic recombination of immune receptors built from immunoglobulin superfamily domains	6.82E-06
GO:0043900	regulation of multi-organism process	6.97E-06
GO:0023051	regulation of signaling	7.19E-06
GO:0002718	regulation of cytokine production involved in immune response	7.46E-06
GO:0050921	positive regulation of chemotaxis	7.46E-06
GO:0065007	biological regulation	8.35E-06
GO:0031325	positive regulation of cellular metabolic process	8.90E-06
GO:0010817	regulation of hormone levels	9.26E-06
GO:0043410	positive regulation of MAPK cascade	9.31E-06
GO:0032800	receptor biosynthetic process	9.40E-06
GO:0045944	positive regulation of transcription by RNA polymerase II	1.11E-05
GO:0042493	response to drug	1.21E-05
GO:2000026	regulation of multicellular organismal development	1.22E-05
GO:0045597	positive regulation of cell differentiation	1.29E-05
GO:0034613	cellular protein localization	1.36E-05
GO:0046883	regulation of hormone secretion	1.36E-05
GO:0007166	cell surface receptor signaling pathway	1.42E-05
GO:0050789	regulation of biological process	1.54E-05
GO:0071310	cellular response to organic substance	1.59E-05
GO:1902115	regulation of organelle assembly	1.83E-05
GO:0015833	peptide transport	1.88E-05
GO:0001938	positive regulation of endothelial cell proliferation	1.94E-05
GO:0033036	macromolecule localization	2.04E-05
GO:0071705	nitrogen compound transport	2.04E-05
GO:0044070	regulation of anion transport	2.10E-05
GO:0045893	positive regulation of transcription, DNA-templated	2.10E-05
GO:0050794	regulation of cellular process	2.17E-05
GO:0051173	positive regulation of nitrogen compound metabolic process	2.20E-05
GO:0050679	positive regulation of epithelial cell proliferation	2.25E-05
GO:0043112	receptor metabolic process	2.38E-05
GO:0010646	regulation of cell communication	2.46E-05
GO:0042035	regulation of cytokine biosynthetic process	2.46E-05
GO:0071702	organic substance transport	2.82E-05
GO:0010557	positive regulation of macromolecule biosynthetic process	2.86E-05

GO:0070887	cellular response to chemical stimulus	2.86E-05
GO:0048468	cell development	2.87E-05
GO:0001817	regulation of cytokine production	2.95E-05
GO:0045935	positive regulation of nucleobase-containing compound metabolic process	2.99E-05
GO:0072359	circulatory system development	2.99E-05
GO:0050678	regulation of epithelial cell proliferation	3.23E-05
GO:0010604	positive regulation of macromolecule metabolic process	3.73E-05
GO:0010628	positive regulation of gene expression	4.08E-05
GO:0043903	regulation of symbiosis, encompassing mutualism through parasitism	4.08E-05
GO:0045073	regulation of chemokine biosynthetic process	4.08E-05
GO:0031328	positive regulation of cellular biosynthetic process	4.55E-05
GO:1903793	positive regulation of anion transport	4.74E-05
GO:0050727	regulation of inflammatory response	5.12E-05
GO:0006935	chemotaxis	5.17E-05
GO:0010033	response to organic substance	5.19E-05
GO:0014070	response to organic cyclic compound	5.27E-05
GO:0050776	regulation of immune response	5.27E-05
GO:0051353	positive regulation of oxidoreductase activity	5.27E-05
GO:0031347	regulation of defense response	5.42E-05
GO:1902017	regulation of cilium assembly	5.53E-05
GO:0034116	positive regulation of heterotypic cell-cell adhesion	5.63E-05
GO:0050729	positive regulation of inflammatory response	5.63E-05
GO:0071495	cellular response to endogenous stimulus	5.63E-05
GO:0009719	response to endogenous stimulus	5.69E-05
GO:0051270	regulation of cellular component movement	5.70E-05
GO:0002889	regulation of immunoglobulin mediated immune response	5.73E-05
GO:0071407	cellular response to organic cyclic compound	5.73E-05
GO:0031503	protein-containing complex localization	5.92E-05
GO:0045428	regulation of nitric oxide biosynthetic process	6.40E-05
GO:0051716	cellular response to stimulus	6.68E-05
GO:0043434	response to peptide hormone	6.89E-05
GO:0031349	positive regulation of defense response	7.23E-05
GO:1905952	regulation of lipid localization	7.27E-05
GO:0030595	leukocyte chemotaxis	7.33E-05
GO:0050896	response to stimulus	7.33E-05
GO:0042130	negative regulation of T cell proliferation	7.40E-05
GO:0008104	protein localization	7.72E-05
GO:0022409	positive regulation of cell-cell adhesion	7.86E-05
GO:0045785	positive regulation of cell adhesion	8.12E-05
GO:0071375	cellular response to peptide hormone stimulus	9.14E-05

GO:0051384	response to glucocorticoid	9.39E-05
GO:0044093	positive regulation of molecular function	9.42E-05
GO:0002637	regulation of immunoglobulin production	9.76E-05
GO:0006810	transport	9.91E-05
GO:0051091	positive regulation of DNA-binding transcription factor activity	9.95E-05
GO:0001819	positive regulation of cytokine production	0.0001
GO:0022607	cellular component assembly	0.00011
GO:0051090	regulation of DNA-binding transcription factor activity	0.00012
GO:0043406	positive regulation of MAP kinase activity	0.00013
GO:1901576	organic substance biosynthetic process	0.00014
GO:0051701	interaction with host	0.00015
GO:0072659	protein localization to plasma membrane	0.00015
GO:0007018	microtubule-based movement	0.00016
GO:0007017	microtubule-based process	0.00017
GO:0007165	signal transduction	0.00017
GO:0007346	regulation of mitotic cell cycle	0.00017
GO:0032642	regulation of chemokine production	0.00017
GO:0032755	positive regulation of interleukin-6 production	0.00017
GO:0061138	morphogenesis of a branching epithelium	0.00017
GO:0006955	immune response	0.00018
GO:0120032	regulation of plasma membrane bounded cell projection assembly	0.00018
GO:0044458	motile cilium assembly	0.00019
GO:0060429	epithelium development	0.00019
GO:0080134	regulation of response to stress	0.00019
GO:0002719	negative regulation of cytokine production involved in immune response	0.0002
GO:0006886	intracellular protein transport	0.0002
GO:0051043	regulation of membrane protein ectodomain proteolysis	0.0002
GO:0051641	cellular localization	0.0002
GO:0048661	positive regulation of smooth muscle cell proliferation	0.00021
GO:0050796	regulation of insulin secretion	0.00021
GO:0050792	regulation of viral process	0.00022
GO:0009725	response to hormone	0.00024
GO:0001568	blood vessel development	0.00025
GO:0002675	positive regulation of acute inflammatory response	0.00025
GO:0033365	protein localization to organelle	0.00025
GO:0045940	positive regulation of steroid metabolic process	0.00025
GO:0051241	negative regulation of multicellular organismal process	0.00025

GO:0010647	positive regulation of cell communication	0.00026
GO:0019221	cytokine-mediated signaling pathway	0.00026
GO:0023056	positive regulation of signaling	0.00026
GO:0044087	regulation of cellular component biogenesis	0.00026
GO:0002708	positive regulation of lymphocyte mediated immunity	0.00028
GO:0007435	salivary gland morphogenesis	0.00028
GO:0002824	positive regulation of adaptive immune response based on somatic recombination of immune receptors built from immunoglobulin superfamily domains	0.00029
GO:0006954	inflammatory response	0.00029
GO:0008285	negative regulation of cell population proliferation	0.00029
GO:0065009	regulation of molecular function	0.0003
GO:0002690	positive regulation of leukocyte chemotaxis	0.00031
GO:0042036	negative regulation of cytokine biosynthetic process	0.00031
GO:0046718	viral entry into host cell	0.00031
GO:0001569	branching involved in blood vessel morphogenesis	0.00033
GO:0045595	regulation of cell differentiation	0.00035
GO:0002874	regulation of chronic inflammatory response to antigenic stimulus	0.00036
GO:0010595	positive regulation of endothelial cell migration	0.00036
GO:0031663	lipopolysaccharide-mediated signaling pathway	0.00036
GO:0032649	regulation of interferon-gamma production	0.00036
GO:0060559	positive regulation of calcidiol 1-monooxygenase activity	0.00036
GO:0071704	organic substance metabolic process	0.00036
GO:0010574	regulation of vascular endothelial growth factor production	0.00038
GO:0051707	response to other organism	0.00038
GO:1903039	positive regulation of leukocyte cell-cell adhesion	0.00038
GO:1904707	positive regulation of vascular smooth muscle cell proliferation	0.00038
GO:0032892	positive regulation of organic acid transport	0.0004
GO:0044057	regulation of system process	0.0004
GO:0002376	immune system process	0.00041
GO:0048856	anatomical structure development	0.00041
GO:0032501	multicellular organismal process	0.00043
GO:0044419	interspecies interaction between organisms	0.00043
GO:0097529	myeloid leukocyte migration	0.00043
GO:0030154	cell differentiation	0.00044
GO:0071345	cellular response to cytokine stimulus	0.00044
GO:0043491	protein kinase B signaling	0.00045

GO:0001990	regulation of systemic arterial blood pressure by hormone	0.00047
GO:0030730	sequestering of triglyceride	0.00047
GO:0051781	positive regulation of cell division	0.00047
GO:0060300	regulation of cytokine activity	0.00047
GO:0031323	regulation of cellular metabolic process	0.00049
GO:0032715	negative regulation of interleukin-6 production	0.00054
GO:0061512	protein localization to cilium	0.00054
GO:1903672	positive regulation of sprouting angiogenesis	0.00054
GO:0019218	regulation of steroid metabolic process	0.00055
GO:0071236	cellular response to antibiotic	0.00055
GO:0002698	negative regulation of immune effector process	0.00056
GO:0035556	intracellular signal transduction	0.00058
GO:0007368	determination of left/right symmetry	0.00059
GO:0048514	blood vessel morphogenesis	0.0006
GO:0009605	response to external stimulus	0.00062
GO:0050715	positive regulation of cytokine secretion	0.00062
GO:0050864	regulation of B cell activation	0.00064
GO:0016032	viral process	0.00066
GO:0045429	positive regulation of nitric oxide biosynthetic process	0.00068
GO:0055025	positive regulation of cardiac muscle tissue development	0.00068
GO:0043902	positive regulation of multi-organism process	0.0007
GO:0035295	tube development	0.00071
GO:0032870	cellular response to hormone stimulus	0.00074
GO:0071347	cellular response to interleukin-1	0.00075
GO:0001818	negative regulation of cytokine production	0.00076
GO:0050778	positive regulation of immune response	0.00077
GO:0060976	coronary vasculature development	0.00079
GO:1903522	regulation of blood circulation	0.00081
GO:0001822	kidney development	0.00082
GO:0007389	pattern specification process	0.00082
GO:0045599	negative regulation of fat cell differentiation	0.00082
GO:0048513	animal organ development	0.00084
GO:0048762	mesenchymal cell differentiation	0.00085
GO:0002009	morphogenesis of an epithelium	0.00086
GO:0019722	calcium-mediated signaling	0.00089
GO:0048754	branching morphogenesis of an epithelial tube	0.00094
GO:0007159	leukocyte cell-cell adhesion	0.00095
GO:0009187	cyclic nucleotide metabolic process	0.00095
GO:0031622	positive regulation of fever generation	0.00095
GO:0045834	positive regulation of lipid metabolic process	0.00095

GO:1903900	regulation of viral life cycle	0.00095
GO:0043269	regulation of ion transport	0.00096
GO:0050777	negative regulation of immune response	0.00097
GO:0030856	regulation of epithelial cell differentiation	0.00099
GO:0019932	second-messenger-mediated signaling	0.001
GO:0042221	response to chemical	0.001
GO:0044260	cellular macromolecule metabolic process	0.001
GO:0060284	regulation of cell development	0.001
GO:0009653	anatomical structure morphogenesis	0.0011
GO:0015031	protein transport	0.0011
GO:0032667	regulation of interleukin-23 production	0.0011
GO:0042045	epithelial fluid transport	0.0011
GO:0043066	negative regulation of apoptotic process	0.0011
GO:0043085	positive regulation of catalytic activity	0.0011
GO:0043170	macromolecule metabolic process	0.0011
GO:0045080	positive regulation of chemokine biosynthetic process	0.0011
GO:0045931	positive regulation of mitotic cell cycle	0.0011
GO:0070372	regulation of ERK1 and ERK2 cascade	0.0011
GO:0072577	endothelial cell apoptotic process	0.0011
GO:0030855	epithelial cell differentiation	0.0012
GO:0043536	positive regulation of blood vessel endothelial cell migration	0.0012
GO:0045840	positive regulation of mitotic nuclear division	0.0012
GO:0051051	negative regulation of transport	0.0012
GO:0051726	regulation of cell cycle	0.0012
GO:0002016	regulation of blood volume by renin-angiotensin	0.0013
GO:0032722	positive regulation of chemokine production	0.0013
GO:0040008	regulation of growth	0.0013
GO:0016043	cellular component organization	0.0014
GO:0030593	neutrophil chemotaxis	0.0014
GO:0033043	regulation of organelle organization	0.0014
GO:0001525	angiogenesis	0.0015
GO:0002696	positive regulation of leukocyte activation	0.0015
GO:0006915	apoptotic process	0.0015
GO:0006937	regulation of muscle contraction	0.0015
GO:0006996	organelle organization	0.0015
GO:0019062	virion attachment to host cell	0.0015
GO:0019229	regulation of vasoconstriction	0.0015
GO:0032308	positive regulation of prostaglandin secretion	0.0015
GO:0035721	intraciliary retrograde transport	0.0015
GO:0046886	positive regulation of hormone biosynthetic process	0.0015

GO:0140056	organelle localization by membrane tethering	0.0015
GO:2001225	regulation of chloride transport	0.0015
GO:0003018	vascular process in circulatory system	0.0016
GO:0014823	response to activity	0.0016
GO:0042325	regulation of phosphorylation	0.0016
GO:0044703	multi-organism reproductive process	0.0016
GO:0046677	response to antibiotic	0.0016
GO:0060627	regulation of vesicle-mediated transport	0.0016
GO:0001780	neutrophil homeostasis	0.0017
GO:0002507	tolerance induction	0.0017
GO:0002923	regulation of humoral immune response mediated by circulating immunoglobulin	0.0017
GO:0042482	positive regulation of odontogenesis	0.0017
GO:0044237	cellular metabolic process	0.0017
GO:0044238	primary metabolic process	0.0017
GO:0046824	positive regulation of nucleocytoplasmic transport	0.0017
GO:0065008	regulation of biological quality	0.0017
GO:1904322	cellular response to forskolin	0.0017
GO:0001776	leukocyte homeostasis	0.0018
GO:0009967	positive regulation of signal transduction	0.0018
GO:0032270	positive regulation of cellular protein metabolic process	0.0018
GO:0032677	regulation of interleukin-8 production	0.0018
GO:0032729	positive regulation of interferon-gamma production	0.0018
GO:0044089	positive regulation of cellular component biogenesis	0.0018
GO:0006952	defense response	0.0019
GO:0032940	secretion by cell	0.0019
GO:0050764	regulation of phagocytosis	0.0019
GO:0061478	response to platelet aggregation inhibitor	0.0019
GO:1901224	positive regulation of NIK/NF-kappaB signaling	0.0019
GO:1902117	positive regulation of organelle assembly	0.0019
GO:1903140	regulation of establishment of endothelial barrier	0.0019
GO:0006807	nitrogen compound metabolic process	0.002
GO:0044130	negative regulation of growth of symbiont in host	0.002
GO:1902930	regulation of alcohol biosynthetic process	0.002
GO:1903531	negative regulation of secretion by cell	0.002
GO:0050731	positive regulation of peptidyl-tyrosine phosphorylation	0.0021
GO:0062012	regulation of small molecule metabolic process	0.0021
GO:0034762	regulation of transmembrane transport	0.0022
GO:0035821	modification of morphology or physiology of other organism	0.0022

GO:0055117	regulation of cardiac muscle contraction	0.0022
GO:1901654	response to ketone	0.0022
GO:0009987	cellular process	0.0023
GO:0035729	cellular response to hepatocyte growth factor stimulus	0.0023
GO:0044126	regulation of growth of symbiont in host	0.0023
GO:0044146	negative regulation of growth of symbiont involved in interaction with host	0.0025
GO:0045408	regulation of interleukin-6 biosynthetic process	0.0025
GO:0050930	induction of positive chemotaxis	0.0025
GO:0001775	cell activation	0.0026
GO:0031100	animal organ regeneration	0.0026
GO:0050870	positive regulation of T cell activation	0.0026
GO:0070374	positive regulation of ERK1 and ERK2 cascade	0.0027
GO:0009214	cyclic nucleotide catabolic process	0.0028
GO:0010638	positive regulation of organelle organization	0.0028
GO:0030949	positive regulation of vascular endothelial growth factor receptor signaling pathway	0.0028
GO:0044144	modulation of growth of symbiont involved in interaction with host	0.0028
GO:0051044	positive regulation of membrane protein ectodomain proteolysis	0.0028
GO:0008016	regulation of heart contraction	0.0029
GO:1901888	regulation of cell junction assembly	0.0029
GO:0002702	positive regulation of production of molecular mediator of immune response	0.003
GO:0010827	regulation of glucose transmembrane transport	0.003
GO:0010888	negative regulation of lipid storage	0.003
GO:0050810	regulation of steroid biosynthetic process	0.003
GO:0051023	regulation of immunoglobulin secretion	0.003
GO:0045069	regulation of viral genome replication	0.0031
GO:0048524	positive regulation of viral process	0.0031
GO:0008015	blood circulation	0.0032
GO:0009966	regulation of signal transduction	0.0032
GO:0019216	regulation of lipid metabolic process	0.0032
GO:0051128	regulation of cellular component organization	0.0032
GO:0022414	reproductive process	0.0033
GO:0032682	negative regulation of chemokine production	0.0033
GO:0045787	positive regulation of cell cycle	0.0033
GO:2000727	positive regulation of cardiac muscle cell differentiation	0.0033
GO:0002726	positive regulation of T cell cytokine production	0.0035
GO:0010829	negative regulation of glucose transmembrane transport	0.0035

GO:1901655	cellular response to ketone	0.0035
GO:1902074	response to salt	0.0035
GO:0007275	multicellular organism development	0.0036
GO:0042102	positive regulation of T cell proliferation	0.0036
GO:0097191	extrinsic apoptotic signaling pathway	0.0037
GO:0016192	vesicle-mediated transport	0.0039
GO:0098542	defense response to other organism	0.0044
GO:0048016	inositol phosphate-mediated signaling	0.0045
GO:0050995	negative regulation of lipid catabolic process	0.0045
GO:0006606	protein import into nucleus	0.0046
GO:0032368	regulation of lipid transport	0.0046
GO:0097305	response to alcohol	0.0046
GO:1901564	organonitrogen compound metabolic process	0.0046
GO:0045926	negative regulation of growth	0.0047
GO:0009059	macromolecule biosynthetic process	0.0048
GO:0035813	regulation of renal sodium excretion	0.0048
GO:0048568	embryonic organ development	0.0049
GO:2001237	negative regulation of extrinsic apoptotic signaling pathway	0.0049
GO:0033138	positive regulation of peptidyl-serine phosphorylation	0.005
GO:0045672	positive regulation of osteoclast differentiation	0.0051
GO:0002683	negative regulation of immune system process	0.0052
GO:0003014	renal system process	0.0052
GO:0009790	embryo development	0.0052
GO:0045930	negative regulation of mitotic cell cycle	0.0052
GO:0051817	modification of morphology or physiology of other organism involved in symbiotic interaction	0.0052
GO:0045321	leukocyte activation	0.0053
GO:0071677	positive regulation of mononuclear cell migration	0.0054
GO:2000778	positive regulation of interleukin-6 secretion	0.0054
GO:0006959	humoral immune response	0.0058
GO:0048585	negative regulation of response to stimulus	0.0058
GO:0050709	negative regulation of protein secretion	0.0058
GO:0042592	homeostatic process	0.0059
GO:0006950	response to stress	0.006
GO:0007568	aging	0.006
GO:0010575	positive regulation of vascular endothelial growth factor production	0.0061
GO:0042981	regulation of apoptotic process	0.0061
GO:0045662	negative regulation of myoblast differentiation	0.0061
GO:0003008	system process	0.0062
GO:0034763	negative regulation of transmembrane transport	0.0063
GO:0035066	positive regulation of histone acetylation	0.0064

GO:0046058	cAMP metabolic process	0.0064
GO:1902105	regulation of leukocyte differentiation	0.0064
GO:0002768	immune response-regulating cell surface receptor signaling pathway	0.0067
GO:0044249	cellular biosynthetic process	0.0067
GO:0048731	system development	0.0068
GO:0060428	lung epithelium development	0.0068
GO:0010613	positive regulation of cardiac muscle hypertrophy	0.0072
GO:0032743	positive regulation of interleukin-2 production	0.0072
GO:0046068	cGMP metabolic process	0.0072
GO:0090023	positive regulation of neutrophil chemotaxis	0.0072
GO:0009636	response to toxic substance	0.0073
GO:0090068	positive regulation of cell cycle process	0.0073
GO:0009154	purine ribonucleotide catabolic process	0.0075
GO:0019538	protein metabolic process	0.0075
GO:0030212	hyaluronan metabolic process	0.0075
GO:0035774	positive regulation of insulin secretion involved in cellular response to glucose stimulus	0.0075
GO:0046330	positive regulation of JNK cascade	0.0075
GO:0048565	digestive tract development	0.0075
GO:0050869	negative regulation of B cell activation	0.0075
GO:0090316	positive regulation of intracellular protein transport	0.0075
GO:1901890	positive regulation of cell junction assembly	0.0075
GO:0051482	positive regulation of cytosolic calcium ion concentration involved in phospholipase C-activating G protein-coupled signaling pathway	0.0078
GO:0071363	cellular response to growth factor stimulus	0.0078
GO:0062013	positive regulation of small molecule metabolic process	0.0079
GO:0006464	cellular protein modification process	0.008
GO:0046887	positive regulation of hormone secretion	0.008
GO:0048871	multicellular organismal homeostasis	0.008
GO:0055023	positive regulation of cardiac muscle tissue growth	0.0081
GO:0090322	regulation of superoxide metabolic process	0.0081
GO:1903829	positive regulation of cellular protein localization	0.0083
GO:0045191	regulation of isotype switching	0.0085
GO:0048566	embryonic digestive tract development	0.0085
GO:0097421	liver regeneration	0.0085
GO:0003012	muscle system process	0.0086
GO:0032965	regulation of collagen biosynthetic process	0.0089
GO:2000249	regulation of actin cytoskeleton reorganization	0.0089
GO:0001505	regulation of neurotransmitter levels	0.009
GO:0051246	regulation of protein metabolic process	0.0091

GO:0019048	modulation by virus of host process	0.0092
GO:1901031	regulation of response to reactive oxygen species	0.0092
GO:0031329	regulation of cellular catabolic process	0.0093
GO:1902107	positive regulation of leukocyte differentiation	0.0093
GO:0002891	positive regulation of immunoglobulin mediated immune response	0.0096
GO:0045823	positive regulation of heart contraction	0.0096
GO:2000785	regulation of autophagosome assembly	0.0096
GO:2001240	negative regulation of extrinsic apoptotic signaling pathway in absence of ligand	0.0096
GO:0016485	protein processing	0.0097
GO:0051092	positive regulation of NF-kappaB transcription factor activity	0.0097
GO:0006904	vesicle docking involved in exocytosis	0.01
GO:0030316	osteoclast differentiation	0.01
GO:0031018	endocrine pancreas development	0.01
GO:0051930	regulation of sensory perception of pain	0.01
GO:0071548	response to dexamethasone	0.01
GO:0045786	negative regulation of cell cycle	0.0101
GO:0019953	sexual reproduction	0.0103
GO:1905515	non-motile cilium assembly	0.0103
GO:0060341	regulation of cellular localization	0.0104
GO:0002521	leukocyte differentiation	0.0105
GO:0003002	regionalization	0.0105
GO:1903034	regulation of response to wounding	0.0106
GO:0000187	activation of MAPK activity	0.0107
GO:0006887	exocytosis	0.0108
GO:0051235	maintenance of location	0.0109
GO:0001959	regulation of cytokine-mediated signaling pathway	0.011
GO:0002790	peptide secretion	0.0114
GO:0042307	positive regulation of protein import into nucleus	0.0116
GO:0048738	cardiac muscle tissue development	0.0116
GO:0050918	positive chemotaxis	0.012
GO:0051248	negative regulation of protein metabolic process	0.0121
GO:0051897	positive regulation of protein kinase B signaling	0.0121
GO:0002639	positive regulation of immunoglobulin production	0.0125
GO:1901135	carbohydrate derivative metabolic process	0.0125
GO:0051171	regulation of nitrogen compound metabolic process	0.0126
GO:0006796	phosphate-containing compound metabolic process	0.0127
GO:0006953	acute-phase response	0.0128

GO:0010718	positive regulation of epithelial to mesenchymal transition	0.0128
GO:0044060	regulation of endocrine process	0.0128
GO:0032970	regulation of actin filament-based process	0.0131
GO:2000112	regulation of cellular macromolecule biosynthetic process	0.0133
GO:0048639	positive regulation of developmental growth	0.0135
GO:0035722	interleukin-12-mediated signaling pathway	0.0137
GO:0045933	positive regulation of muscle contraction	0.0137
GO:0050766	positive regulation of phagocytosis	0.0137
GO:0044283	small molecule biosynthetic process	0.014
GO:0032147	activation of protein kinase activity	0.0141
GO:0032757	positive regulation of interleukin-8 production	0.0141
GO:0006355	regulation of transcription, DNA-templated	0.0142
GO:0048646	anatomical structure formation involved in morphogenesis	0.0143
GO:0051640	organelle localization	0.0144
GO:0010468	regulation of gene expression	0.0145
GO:0035094	response to nicotine	0.0145
GO:0050832	defense response to fungus	0.0145
GO:0032268	regulation of cellular protein metabolic process	0.0146
GO:0051130	positive regulation of cellular component organization	0.0149
GO:1901136	carbohydrate derivative catabolic process	0.0153
GO:0019219	regulation of nucleobase-containing compound metabolic process	0.0155
GO:0080090	regulation of primary metabolic process	0.0162
GO:0060688	regulation of morphogenesis of a branching structure	0.0163
GO:0030278	regulation of ossification	0.0165
GO:0002520	immune system development	0.0168
GO:0006979	response to oxidative stress	0.0172
GO:0071385	cellular response to glucocorticoid stimulus	0.0173
GO:0000302	response to reactive oxygen species	0.0182
GO:0051052	regulation of DNA metabolic process	0.0183
GO:1901215	negative regulation of neuron death	0.0187
GO:0003006	developmental process involved in reproduction	0.0189
GO:0014032	neural crest cell development	0.0189
GO:0050710	negative regulation of cytokine secretion	0.0189
GO:0060255	regulation of macromolecule metabolic process	0.0189
GO:0022008	neurogenesis	0.0192
GO:0030858	positive regulation of epithelial cell differentiation	0.0192
GO:1901137	carbohydrate derivative biosynthetic process	0.0192
GO:1903902	positive regulation of viral life cycle	0.0192

GO:0097237	cellular response to toxic substance	0.0194
GO:0030072	peptide hormone secretion	0.0198
GO:0046888	negative regulation of hormone secretion	0.0198
GO:0071356	cellular response to tumor necrosis factor	0.0198
GO:0048732	gland development	0.0202
GO:0002223	stimulatory C-type lectin receptor signaling pathway	0.0208
GO:0048015	phosphatidylinositol-mediated signaling	0.0208
GO:0050891	multicellular organismal water homeostasis	0.0208
GO:0001558	regulation of cell growth	0.0212
GO:0006468	protein phosphorylation	0.0214
GO:0043583	ear development	0.0214
GO:0070301	cellular response to hydrogen peroxide	0.0218
GO:2000027	regulation of animal organ morphogenesis	0.0222
GO:1901992	positive regulation of mitotic cell cycle phase transition	0.0224
GO:0009612	response to mechanical stimulus	0.0228
GO:0098876	vesicle-mediated transport to the plasma membrane	0.0228
GO:0044706	multi-multicellular organism process	0.0231
GO:0048010	vascular endothelial growth factor receptor signaling pathway	0.0233
GO:0032868	response to insulin	0.0235
GO:0010648	negative regulation of cell communication	0.0236
GO:0001837	epithelial to mesenchymal transition	0.0239
GO:0023057	negative regulation of signaling	0.0239
GO:0009150	purine ribonucleotide metabolic process	0.0248
GO:0008283	cell population proliferation	0.0249
GO:0030279	negative regulation of ossification	0.0255
GO:0043507	positive regulation of JUN kinase activity	0.0255
GO:0045596	negative regulation of cell differentiation	0.0257
GO:0010564	regulation of cell cycle process	0.0259
GO:0034103	regulation of tissue remodeling	0.0267
GO:0030098	lymphocyte differentiation	0.0269
GO:1900407	regulation of cellular response to oxidative stress	0.0273
GO:0008306	associative learning	0.0278
GO:0030100	regulation of endocytosis	0.0278
GO:0032943	mononuclear cell proliferation	0.0285
GO:0002429	immune response-activating cell surface receptor signaling pathway	0.0292
GO:0071260	cellular response to mechanical stimulus	0.0297
GO:0055007	cardiac muscle cell differentiation	0.0304
GO:0048519	negative regulation of biological process	0.0307

GO:0032088	negative regulation of NF-kappaB transcription factor activity	0.031
GO:0050871	positive regulation of B cell activation	0.031
GO:0090066	regulation of anatomical structure size	0.0321
GO:1901362	organic cyclic compound biosynthetic process	0.0323
GO:0045778	positive regulation of ossification	0.033
GO:0042742	defense response to bacterium	0.0341
GO:0007193	adenylate cyclase-inhibiting G protein-coupled receptor signaling pathway	0.0342
GO:0071158	positive regulation of cell cycle arrest	0.0342
GO:0051345	positive regulation of hydrolase activity	0.0344
GO:0001657	ureteric bud development	0.0348
GO:0110020	regulation of actomyosin structure organization	0.0348
GO:0044772	mitotic cell cycle phase transition	0.0351
GO:0090287	regulation of cellular response to growth factor stimulus	0.0351
GO:0009953	dorsal/ventral pattern formation	0.0352
GO:0097327	response to antineoplastic agent	0.0352
GO:0009628	response to abiotic stimulus	0.0357
GO:0031023	microtubule organizing center organization	0.0357
GO:0050878	regulation of body fluid levels	0.0357
GO:0010720	positive regulation of cell development	0.0375
GO:0006885	regulation of pH	0.0379
GO:0021782	glial cell development	0.0379
GO:0048609	multicellular organismal reproductive process	0.038
GO:0007267	cell-cell signaling	0.0386
GO:0042552	myelination	0.0392
GO:0120034	positive regulation of plasma membrane bounded cell projection assembly	0.0392
GO:0007030	Golgi organization	0.0405
GO:0097711	ciliary basal body-plasma membrane docking	0.0405
GO:0048699	generation of neurons	0.0409
GO:0006275	regulation of DNA replication	0.0411
GO:0010558	negative regulation of macromolecule biosynthetic process	0.0411
GO:0044281	small molecule metabolic process	0.0411
GO:0009895	negative regulation of catabolic process	0.0414
GO:0006493	protein O-linked glycosylation	0.0416
GO:0007423	sensory organ development	0.0428
GO:0002250	adaptive immune response	0.0437
GO:0010506	regulation of autophagy	0.0459
GO:0043200	response to amino acid	0.0461
GO:0001666	response to hypoxia	0.0466
GO:0007399	nervous system development	0.0472

GO:0010876	lipid localization	0.0486
GO:0031327	negative regulation of cellular biosynthetic process	0.0487
GO:0034654	nucleobase-containing compound biosynthetic process	0.0498
Molecular function (GO)		
GO:0005125	cytokine activity	1.56E-06
GO:0048018	receptor ligand activity	1.61E-05
GO:0005126	cytokine receptor binding	4.26E-05
GO:0005515	protein binding	0.00015
GO:0005488	binding	0.0016
GO:0030551	cyclic nucleotide binding	0.0017
GO:0005102	signaling receptor binding	0.0018
GO:0098772	molecular function regulator	0.0018
GO:0070851	growth factor receptor binding	0.0028
GO:0046790	virion binding	0.0035
GO:0008083	growth factor activity	0.005
GO:0016018	cyclosporin A binding	0.0069
GO:0030553	cGMP binding	0.0069
GO:0001618	virus receptor activity	0.007
GO:0004114	3',5'-cyclic-nucleotide phosphodiesterase activity	0.0162
GO:0045505	dynein intermediate chain binding	0.0183
GO:0097367	carbohydrate derivative binding	0.0183
GO:0001103	RNA polymerase II repressing transcription factor binding	0.0186
GO:0001664	G protein-coupled receptor binding	0.0186
GO:0051959	dynein light intermediate chain binding	0.0186
GO:0003774	motor activity	0.0209
GO:0017111	nucleoside-triphosphatase activity	0.0297
GO:0016787	hydrolase activity	0.0361
GO:0003823	antigen binding	0.0449
GO:0032555	purine ribonucleotide binding	0.0466
Cellular component (GO)		
GO:0097542	ciliary tip	8.64E-06
GO:0005615	extracellular space	0.00024
GO:0005813	centrosome	0.00024
GO:0005815	microtubule organizing center	0.00024
GO:0036064	ciliary basal body	0.00024
GO:0005929	cilium	0.00033
GO:0005868	cytoplasmic dynein complex	0.00035
GO:0015630	microtubule cytoskeleton	0.00077
GO:0030990	intraciliary transport particle	0.00077
GO:0005576	extracellular region	0.0019

GO:0120025	plasma membrane bounded cell projection	0.0022
GO:0009986	cell surface	0.0042
GO:0012505	endomembrane system	0.0042
GO:0031514	motile cilium	0.0043
GO:0099503	secretory vesicle	0.0043
GO:0032838	plasma membrane bounded cell projection cytoplasm	0.0048
GO:0005886	plasma membrane	0.0089
GO:0030141	secretory granule	0.0089
GO:0030992	intraciliary transport particle B	0.0089
GO:0005856	cytoskeleton	0.0098
GO:0005930	axoneme	0.0112
GO:0097546	ciliary base	0.0133
GO:0005814	centriole	0.0153
GO:0031410	cytoplasmic vesicle	0.0153
GO:0032991	protein-containing complex	0.0199
GO:0055037	recycling endosome	0.0267
GO:0030136	clathrin-coated vesicle	0.0296
GO:0045177	apical part of cell	0.0339
GO:0099512	supramolecular fiber	0.0341
GO:0005622	intracellular	0.0348
GO:0005874	microtubule	0.0348
GO:0035869	ciliary transition zone	0.0348
GO:0099513	polymeric cytoskeletal fiber	0.0377
GO:0031093	platelet alpha granule lumen	0.0416
GO:0009897	external side of plasma membrane	0.0453
GO:0055038	recycling endosome membrane	0.0466

Local network cluster (String)

CL:20274	mixed, incl. Ciliopathy, and dynein complex	2.57E-09
CL:20424	intraciliary transport involved in cilium assembly, and BBSome	9.10E-08
CL:20426	intraciliary transport involved in cilium assembly	7.06E-07
CL:20428	intraciliary transport particle	7.71E-06
CL:20560	RFX DNA-binding domain, and multi-ciliated epithelial cell differentiation	0.00011
CL:20562	RFX1 transcription activation region, and Poxvirus D5 protein-like	0.0015
CL:22274	cGMP catabolic process, and Haem-NO-binding	0.0042
CL:20430	intraciliary transport particle B	0.0086
CL:3903	Jak-STAT signaling pathway	0.0271

KEGG pathways

hsa04933	AGE-RAGE signaling pathway in diabetic complications	2.30E-09
hsa05144	Malaria	4.11E-08
hsa05321	Inflammatory bowel disease (IBD)	1.28E-07
hsa05323	Rheumatoid arthritis	5.31E-07
hsa04060	Cytokine-cytokine receptor interaction	7.11E-07
hsa04657	IL-17 signaling pathway	7.11E-07
hsa05142	Chagas disease (American trypanosomiasis)	7.11E-07
hsa05146	Amoebiasis	7.11E-07
hsa05152	Tuberculosis	7.11E-07
hsa05133	Pertussis	4.92E-06
hsa05143	African trypanosomiasis	9.47E-06
hsa04659	Th17 cell differentiation	1.87E-05
hsa04962	Vasopressin-regulated water reabsorption	2.08E-05
hsa05134	Legionellosis	4.17E-05
hsa05166	HTLV-I infection	6.74E-05
hsa04932	Non-alcoholic fatty liver disease (NAFLD)	8.36E-05
hsa05140	Leishmaniasis	9.11E-05
hsa04218	Cellular senescence	9.22E-05
hsa04022	cGMP-PKG signaling pathway	9.84E-05
hsa05164	Influenza A	0.00012
hsa05132	Salmonella infection	0.00015
hsa01523	Antifolate resistance	0.00023
hsa05200	Pathways in cancer	0.00026
hsa04620	Toll-like receptor signaling pathway	0.00027
hsa04668	TNF signaling pathway	0.00031
hsa05332	Graft-versus-host disease	0.00031
hsa04380	Osteoclast differentiation	0.0005
hsa04672	Intestinal immune network for IgA production	0.0005
hsa04926	Relaxin signaling pathway	0.00055
hsa05161	Hepatitis B	0.00074
hsa04010	MAPK signaling pathway	0.001
hsa04621	NOD-like receptor signaling pathway	0.0012
hsa04924	Renin secretion	0.0012
hsa04927	Cortisol synthesis and secretion	0.0012
hsa05202	Transcriptional misregulation in cancer	0.0012
hsa05168	Herpes simplex infection	0.0015
hsa05410	Hypertrophic cardiomyopathy (HCM)	0.002
hsa04350	TGF-beta signaling pathway	0.0021
hsa04540	Gap junction	0.0024
hsa05414	Dilated cardiomyopathy (DCM)	0.0024
hsa04064	NF-kappa B signaling pathway	0.0027
hsa04640	Hematopoietic cell lineage	0.0027

hsa04972	Pancreatic secretion	0.0027
hsa04660	T cell receptor signaling pathway	0.003
hsa04614	Renin-angiotensin system	0.0033
hsa05145	Toxoplasmosis	0.0038
hsa04114	Oocyte meiosis	0.0044
hsa05310	Asthma	0.0044
hsa04110	Cell cycle	0.005
hsa04068	FoxO signaling pathway	0.0057
hsa05020	Prion diseases	0.0057
hsa05162	Measles	0.0058
hsa05418	Fluid shear stress and atherosclerosis	0.0058
hsa05330	Allograft rejection	0.006
hsa04072	Phospholipase D signaling pathway	0.007
hsa04940	Type I diabetes mellitus	0.0074
hsa04934	Cushing's syndrome	0.0079
hsa04217	Necroptosis	0.008
hsa05010	Alzheimer's disease	0.0098
hsa05110	Vibrio cholerae infection	0.0098
hsa00230	Purine metabolism	0.0103
hsa05014	Amyotrophic lateral sclerosis (ALS)	0.0103
hsa04020	Calcium signaling pathway	0.011
hsa04923	Regulation of lipolysis in adipocytes	0.011
hsa05167	Kaposi's sarcoma-associated herpesvirus infection	0.0113
hsa04024	cAMP signaling pathway	0.0132
hsa04623	Cytosolic DNA-sensing pathway	0.0141
hsa04622	RIG-I-like receptor signaling pathway	0.0175
hsa04976	Bile secretion	0.0177
hsa04971	Gastric acid secretion	0.0179
hsa04970	Salivary secretion	0.0247
hsa05032	Morphine addiction	0.027
hsa04750	Inflammatory mediator regulation of TRP channels	0.0272
hsa01522	Endocrine resistance	0.0274
hsa04713	Circadian entrainment	0.0274
hsa04914	Progesterone-mediated oocyte maturation	0.0274
hsa04925	Aldosterone synthesis and secretion	0.0274
hsa05322	Systemic lupus erythematosus	0.0274
hsa04066	HIF-1 signaling pathway	0.0283
hsa04916	Melanogenesis	0.0283
hsa04931	Insulin resistance	0.0324
hsa04724	Glutamatergic synapse	0.0349
hsa04270	Vascular smooth muscle contraction	0.0385
hsa04152	AMPK signaling pathway	0.0387
hsa04611	Platelet activation	0.04

hsa04650	Natural killer cell mediated cytotoxicity	0.0401
hsa05160	Hepatitis C	0.0438
hsa04371	Apelin signaling pathway	0.0446
hsa04915	Estrogen signaling pathway	0.0446
hsa04261	Adrenergic signaling in cardiomyocytes	0.0472
Reactome pathways		
HSA-6785807	Interleukin-4 and Interleukin-13 signaling	3.96E-07
HSA-5617833	Cilium Assembly	4.23E-07
HSA-6783783	Interleukin-10 signaling	3.24E-06
HSA-5620924	Intraflagellar transport	3.91E-06
HSA-162582	Signal Transduction	7.55E-06
HSA-449147	Signaling by Interleukins	5.27E-05
HSA-168256	Immune System	0.0038
HSA-5621481	C-type lectin receptors (CLRs)	0.0038
HSA-110056	MAPK3 (ERK1) activation	0.005
HSA-2025928	Calcineurin activates NFAT	0.005
HSA-111885	Opioid Signalling	0.0121
HSA-2565942	Regulation of PLK1 Activity at G2/M Transition	0.0122
HSA-418457	cGMP effects	0.0122
HSA-5620912	Anchoring of the basal body to the plasma membrane	0.0154
HSA-180024	DARPP-32 events	0.017
HSA-5610787	Hedgehog 'off' state	0.0171
HSA-114452	Activation of BH3-only proteins	0.0218
HSA-114608	Platelet degranulation	0.0235
HSA-8950505	Gene and protein expression by JAK-STAT signaling after Interleukin-12 stimulation	0.0313
HSA-2559583	Cellular Senescence	0.0386
HSA-5620920	Cargo trafficking to the periciliary membrane	0.044
HSA-109582	Hemostasis	0.0491
HSA-375276	Peptide ligand-binding receptors	0.0491
Annotated Keyword (Uniprot)		
KW-0970	Cilium biogenesis/degradation	1.43E-07
KW-0964	Secreted	1.82E-05
KW-0202	Cytokine	5.24E-05
KW-0969	Cilium	0.00027
KW-0243	Dynein	0.00093
KW-0497	Mitogen	0.0014
KW-1186	Ciliopathy	0.0028
KW-0142	cGMP-binding	0.0029
KW-1183	Host cell receptor for virus entry	0.0043
KW-0325	Glycoprotein	0.0105

KW-0206	Cytoskeleton	0.0128
KW-0945	Host-virus interaction	0.0128
KW-0966	Cell projection	0.0128
KW-1015	Disulfide bond	0.0128
KW-0339	Growth factor	0.015
KW-0505	Motor protein	0.015
KW-0449	Lipoprotein	0.025
KW-1003	Cell membrane	0.025
KW-0519	Myristate	0.0296
KW-0550	Obesity	0.0358
KW-0010	Activator	0.0439
Protein domain (SMART)		
SM00471	Metal dependent phosphohydrolases with conserved 'HD' motif.	0.0352
SM00382	ATPases associated with a variety of cellular activities	0.0418

GO: gene ontology; KEGG: Kyoto encyclopedia of gene and genomes; pfam: Protein family database; SMART: Simple modular architecture research tool.

Table S3: List of differentially expressed transcripts related to mucociliary clearance machinery in SARS-CoV-2 infected patients relative to healthy individuals.

Transcript	Log2 fold change (L2FC)	Adjusted p-value (*padj*)
cdk1	-1.0488	0.29
mcm3	-1.0490	0.10
dynll1	-0.7203	0.42
rab8A	0.8100	0.43
atmin	-0.0939	0.93
ift20	-0.1036	NA
ift88	-0.4510	NA
bbs1	-0.1335	NA
dync2h1	-1.0211	0.46
dync2li1	-0.6204	NA
rfx3	-1.0636	0.28
foxJ1	-0.5372	0.61
mcidas	-0.2076	NA
adcy3	0.0428	NA
pde4B	2.1212	0.12
pde5A	-0.1159	NA
prkg2	-1.1511	NA
cxcl8	1.3257	0.40
IL1B	0.3393	0.85
IL10	0.0791	NA
IL17A	2.6590	NA
IL6	-2.8900	NA

tnf	-1.0534	NA
edn1	0.6971	NA
cftr	-0.5268	NA
agtR1	-0.3530	NA
muc5AC	-0.3723	0.69
ace2	1.2246	NA
tmprss2	-1.4190	NA
ppia	-1.2904	0.12
ppp3r1	-0.2516	0.82

References

1. Szklarczyk, D.; Morris, J.H.; Cook, H.; Kuhn, M.; Wyder, S.; Simonovic, M.; Santos, A.; Doncheva, N.T.; Roth, A.; Bork, P., et al. The STRING database in 2017: quality-controlled protein-protein association networks, made broadly accessible. *Nucleic Acids Res* 2017, *45*, D362-D368.
2. Benjamini, Y.; Hochberg, Y. Controlling the False Discovery Rate: A Practical and Powerful Approach to Multiple Testing. *Journal of the Royal Statistical Society: Series B (Methodological)* 1995, *57*, 289-300.
3. Shannon, P.; Markiel, A.; Ozier, O.; Baliga, N.S.; Wang, J.T.; Ramage, D.; Amin, N.; Schwikowski, B.; Ideker, T. Cytoscape: a software environment for integrated models of biomolecular interaction networks. *Genome Res* 2003, *13*, 2498-2504.
4. Brandes, U. A faster algorithm for betweenness centrality*. *The Journal of Mathematical Sociology* 2001, *25*, 163-177.
5. Enright, A.J.; Van Dongen, S.; Ouzounis, C.A. An efficient algorithm for large-scale detection of protein families. *Nucleic Acids Res* 2002, *30*, 1575-1584.
6. Pratt, D.; Chen, J.; Welker, D.; Rivas, R.; Pillich, R.; Rynkov, V.; Ono, K.; Miello, C.; Hicks, L.; Szalma, S., et al. NDEx, the Network Data Exchange. *Cell Syst* 2015, *1*, 302-305.
7. Pratt, D.; Chen, J.; Pillich, R.; Rynkov, V.; Gary, A.; Demchak, B.; Ideker, T. NDEx 2.0: A Clearinghouse for Research on Cancer Pathways. *Cancer Res* 2017, *77*, e58-e61.
8. Pillich, R.T.; Chen, J.; Rynkov, V.; Welker, D.; Pratt, D. NDEx: A Community Resource for Sharing and Publishing of Biological Networks. *Methods Mol Biol* 2017, *1558*, 271-301.
9. Perfetto, L.; Pastrello, C.; Del-Toro, N.; Duesbury, M.; Iannuccelli, M.; Kotlyar, M.; Licata, L.; Meldal, B.; Panneerselvam, K.; Panni, S., et al. The IMEx Coronavirus interactome: an evolving map of Coronaviridae-Host molecular interactions. *bioRxiv* 2020.
10. Lieberman, N.A.P.; Peddu, V.; Xie, H.; Shrestha, L.; Huang, M.L.; Mears, M.C.; Cajimat, M.N.; Bente, D.A.; Shi, P.Y.; Bovier, F., et al. In vivo antiviral host transcriptional response to SARS-CoV-2 by viral load, sex, and age. *PLoS Biol* 2020, *18*, e3000849.
11. Love, M.I.; Huber, W.; Anders, S. Moderated estimation of fold change and dispersion for RNA-seq data with DESeq2. *Genome Biol* 2014, *15*, 550.
12. Team, R. *RStudio: Integrated Development for R. RStudio,*, 2020.
